# Distinct timescales dissociate spontaneous thought dimensions

**DOI:** 10.1073/pnas.2427088122

**Published:** 2025-09-17

**Authors:** Jingyu Hua, Xianliang Ge, Min Dou, Yuqi Zhang, Liezhong Ge, Stuart Fogel, Jianfeng Zhang, Georg Northoff

**Affiliations:** ^a^Center for Brain Disorders and Cognitive Sciences, School of Psychology, Shenzhen University, Shenzhen, Guangdong 518060, China; ^b^Center for Psychological Sciences, Zhejiang University, Hangzhou, Zhejiang 310028, China; ^c^Department of Psychology, Faculty of Social Sciences, University of Ottawa, Ottawa, ON K1N 6N5, Canada; ^d^Mind, Brain Imaging and Neuroethics Research Unit, The Royal’s Institute of Mental Health Research, University of Ottawa, Ottawa, ON K1Z 7K4, Canada; ^e^School of Psychology, Shaanxi Normal University, Xian, Shaanxi 710062, China

**Keywords:** task relatedness, thought orientation, timescale, neural oscillation, midn wandering

## Abstract

Our spontaneous thoughts encompass various dimensions, such as task-relatedness (off vs. on task), and thought orientation (internal vs. external). However, their distinction remains unclear. Our study addresses this issue by focusing on their timescales at both the behavioral level (using fast and slow finger tapping) and the neural level (using EEG) using two independent datasets (N = 84 and 35). Behavioral results revealed a double dissociation: Task-relatedness was linked to fast tapping only, whereas thought orientation was associated with slow tapping only. At the neural level, we assessed topographic similarity in EEG to quantify the temporal influence of past neural activity on current ones. Task-relatedness modulated topographic similarity only during fast tapping, while thought orientation did so only during slow tapping. Critically, topographic similarity was phase-based, as shown by its correlation with phase-locking value and the loss of associations with thought after phase-shuffling. This indicates that the neural signatures of both thought dimensions are strongly phase-dependent. Finally, we demonstrate that nonlinearity plays a distinct role mediating the impact of different timescales (slow and fast finger tapping) on spontaneous thoughts at both behavioral (precision error) and neural levels (topographic similarity). Overall, these results demonstrate that task-relatedness is associated with short timescales, whereas thought orientation is associated with long timescales. This highlights how distinct temporal dynamics shape different spontaneous thought dimensions on both their behavioral and neural features.

## Significance

Spontaneous thought is inherently multidimensional, yet how its core dimensions—such as task-relatedness and thought orientation—differ in their neural and behavioral signatures remains unclear. By combining behavioral tapping paradigms with EEG across two independent datasets, we reveal a double dissociation: Task-relatedness operates over short timescales, while thought orientation spans longer timescales. These distinctions are mirrored in brain activity, where phase-based topographic similarity selectively tracks each thought dimension within its corresponding timescale. Our findings provide compelling evidence that distinct dimensions of spontaneous thought are shaped by dissociable temporal dynamics, offering timescales as a framework for understanding the neural architecture of spontaneous thought.

Humans exhibit a multitude of thoughts that are highly multidimensional. One dimension of thought, namely spontaneous thought, is commonly described as the experience of “mind wandering” (1, 2). Mind wandering can include thoughts during a task that remain unrelated to the task at hand, i.e., “off-task” thoughts. A wealth of functional brain imaging work has distinguished these from “on-task” thoughts. On- and off-task thoughts can thus be considered two ends of the spectrum of thought “task-relatedness”. Yet another dimension of thought concerns the “orientation*”* of its content, that is, whether it is directed internally toward the self (i.e., thoughts about events related to the inner self), or, externally toward the environment (i.e., thoughts about features and events in the outer environment). Recent studies suggest that thought orientation and task-relatedness could be distinct dimensions, differing in their neural correlates and temporal dynamics (3–5). The extent to which these dimensions are dissociable at the behavioral and neural levels in terms of their temporal structure and processing mechanisms remains unclear. Understanding the temporal and spatial dynamics of thought, and linking them at the neural and behavioral level will provide novel understanding of the temporal shaping of our thought in terms of their timescales. This would add additional support to what recently has been introduced as Spatial-Temporal Neuroscience (6–8).

One fundamental and unresolved question about the nature of spontaneous human thought is whether off-task thoughts are equivalent to internally oriented thoughts, and, on the other hand, whether on-task thoughts are equivalent to externally oriented thoughts? Traditionally, the two dimensions of “task-relatedness” (on vs. off-task) and “thought orientation” (external vs. internal) thoughts were presumed as equivalent (9–11). However, emerging empirical and neuroimaging evidence suggests a functional and anatomical dissociation between them, thus suggesting that they should be considered separately. For example, task-relatedness measures the extent to which one’s thoughts are focused on the task-at-hand (1, 2, 12), which can range on a continuum from completely focused and engrossed in the task (“on-task”), to absent-minded, wandering thoughts (“off-task”). By contrast, thought orientation measures whether the thought content is focused more on the external environment, or, at the other end of the continuum, focused on the self, and thus, internally on one’s own mental state (5, 13–15). Furthermore, the extremes of the spectrum of the task-relatedness and thought orientation dimensions suggest that they are dissociable in terms of their functional neuroanatomical differences in the recruited brain regions, e.g., sensory vs. prefrontal regions, and their neuronal timescales, fast vs. slow (4, 5, 14). Again, supporting the notion that these are separate dimensions. However, investigation directly comparing the two spontaneous thought dimensions remains to be conducted.

Given the yet unclear characterization and differentiation of task-relatedness and thought orientation, as potentially distinct thought dimensions, the goal of this study is to directly compare them to one another by investigating their interrelationship and possible distinction in terms of both behavioral and neural correlates. Our focus is especially on their timescales which, broadly speaking, refer to the duration of the thought process that could be either longer, or shorter (2, 4). Based on previous findings (3, 5, 16), we hypothesize that the two thought dimensions can be distinguished by their timescales, and thus by their durations at both the behavioral and neural levels.

Potential differences in timescales between thought dimensions may offer a means to distinguish them at both the behavioral and neural levels. Prior work suggests that thought orientation (i.e., internal vs. external thoughts) unfolds over longer durations than task-relatedness (i.e., on-task vs. off-task thoughts), indicating a longer timescale for orientation compared to task-relatedness. Accordingly, these potential timescale differences may be useful to distinguish the two thought dimensions on both behavioral and neural grounds (4, 5). To examine this at the behavioral level, we employed the finger tapping paradigm, a widely used method for indirectly indexing ongoing thought through motor variability. Tapping variability has been shown to correlate with fluctuations in attention and mind-wandering: Higher variability reflects off-task thought, while lower variability reflects on-task engagement (3, 17, 18). Further, high variability of spontaneous finger tapping has been found to be associated with reduced attention, indicating off-task thought, whereas low variability in finger tapping relates to on-task thought (17). Moreover, variability can also serve as index of speed and thus timescales (19). Therefore, we used the variability of slow and fast finger tapping to investigate the timescales of different thought dimensions. In this study, we used precision error (PE) to quantify tapping variability and investigate whether PE during slow and fast finger tapping, as a behavioral proxy of timescales, differentially correlates with thought dimensions. Specifically, we hypothesize that PE will be more strongly associated with task-relatedness during fast tapping—reflecting shorter timescales—and with thought orientation during slow tapping, which engages longer timescales.

What about the distinct neural correlates of task-relatedness and thought orientation dimensions? In terms of brain dynamics, timescales can be measured from how EEG activity at an earlier time point influences neural activity at later time point. This can be measured using “topographic similarity”, which quantifies the resemblance between the topography at one time point with those at subsequent time points (20, 21). More precisely, topographic similarity measures the extent to which the spatial configurations of EEG derivations at past time points influence their distribution at the present time point (20, 21). The higher the topographic similarity, the stronger the influence of the past on the present, and the higher the degree of temporal integration of past and present inputs across a given duration (i.e., timescale) (22, 23). Topographic similarity can thus be considered a neural proxy for neural duration, which therefore reflects the timescale of an underlying neural process. We hypothesize that topographic similarity during fast tapping (short neural timescales) will correlate more strongly with task-relatedness, while topographic similarity during slow tapping (longer timescales) will be more sensitive to thought orientation. Crucially, our study goes beyond linear associations and tests whether these relationships are mediated by nonlinear mechanisms. Mounting evidence suggests that the mapping between thought and neural activity may be nonlinear—particularly for intermediate cognitive states (e.g., ambiguous task engagement) that suppress or mask linear effects (24–27). To examine this, we employed generalized additive models (GAMs) and Bayesian mediation analyses, allowing us to differentiate direct linear effects from indirect nonlinear influences.

Finally, we also investigate the neural mechanisms underlying these timescales by examining whether phase-based neural synchrony (PLV) underlies the observed patterns. Unlike traditional power-based measures, phase dynamics reflect large-scale network coordination and temporal coupling between distant brain regions (28, 29). We predict that topographic similarity is fundamentally phase-based, and that the modulation of thought by neural timescales is grounded in phase synchrony rather than local amplitude fluctuations.

**Fig. 1.**
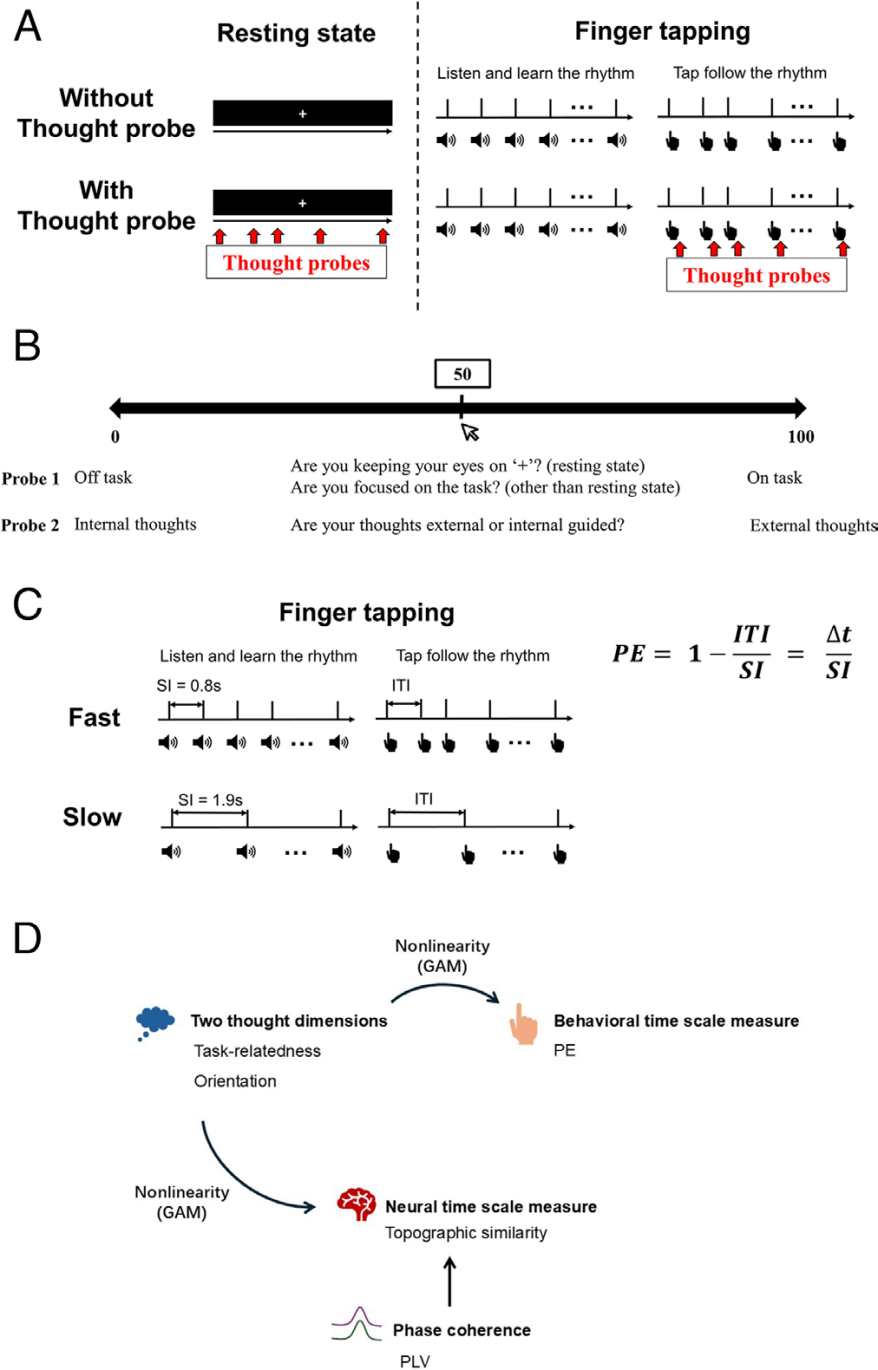
Schema of the experimental paradigm. (*A*) Overall schema of experiment. The procedure included two tasks: resting state and finger tapping. Participants first completed a 10-min resting session with eyes open, fixating on a cross and staying relaxed. In the finger tapping task, they first heard 11 tones and then tapped to the learned rhythm for 10 min. Both resting and tapping tasks (fast and slow) were repeated twice—once with and once without thought probes. Thought probes asked participants to rate their two thought dimensions on a 0 to 100 VAS (*B*) Thought probes. 20 thought probes were included in each task with intervals randomized from 5 s to 65 s, jittered in 10 s intervals. Each thought probe contained two questions, one about off vs. on task thought, the other about internal vs. external thoughts. (*C*) Schema of finger tapping and calculation of PE. There were two distinct speeds for the finger tapping task as indicated by the ITI: fast ITI = 0.8 s, and slow ITI = 1.9 s. The PE was calculated to capture the deviation between the mean of the real ITI and the presumed standard interval (SI) by using the ratio between them: PE = 1-ITI/SI. (*D*) Schematic illustration of the analytical framework of nonlinearity and phase basis of topographic similarity. Note: ITI: intertapping interval; SI: presumed standard interval; PE: precision error.

In sum, our study aims to disentangle the temporal dynamics of two fundamental dimensions of spontaneous thought—task-relatedness and thought orientation—across cognitive, behavioral, and neural domains. By combining finger tapping, EEG-based topographic similarity, phase synchrony, and advanced nonlinear modeling, we seek to establish that these two dimensions are governed by dissociable timescales and mechanistically distinct neural processes (Fig. 1*D*).

## Method

### Participants.

Dataset 1 included 84 right-handed adults (41 female; 18 to 30 y, M = 21.0, SD = 2.3) after excluding 2 for poor EEG quality. Protocols were approved by ethics boards at Shenzhen University and University of Ottawa. Dataset 2 included 35 right-handed adults (11 female; 18 to 28 y, M = 22.6, SD = 2.4). Protocols were approved by ethics boards at Zhejiang University and University of Ottawa. All had normal or corrected vision, no history of neurological/psychiatric conditions, and provided written informed consent.

### Procedures.

Participants completed a 10-min eyes-open resting state followed by finger tapping tasks (Fig. 1*A*). In the tapping task, they first heard 11 auditory tones and then tapped to the learned rhythm for 10 min. Both resting and tapping tasks (at fast and slow speeds) were repeated twice—once with and once without thought probes (Fig. 1*A*) (30). Based on prior studies, the spontaneous tapping tempo is ~0.6 s (30, 31), with best motor performance at this interval. To balance proximity to spontaneous tempo and inclusion of slow EEG rhythms, fast tapping used a standard interval (SI) of 0.8 s, and slow tapping used 1.9 s to avoid harmonic overlap (Fig. 1*C*, also see *SI Appendix* for details)

### Thought Probes.

Twenty thought probes were presented per session, with randomized interprobe intervals (5 to 65 s, jittered by 10 s, Fig. 1*A*). Each probe asked participants to rate their momentary thought along two dimensions using a 0 to 100 visual analog scale (VAS): task-relatedness (off/on-task) and orientation (internal/external). Different phrasings were used in resting and task contexts to suit task relevance (Fig. 1*B*). Higher scores indicated more on-task and externally oriented states. See *SI Appendix* for details.

### PE.

**Fig. 2.**
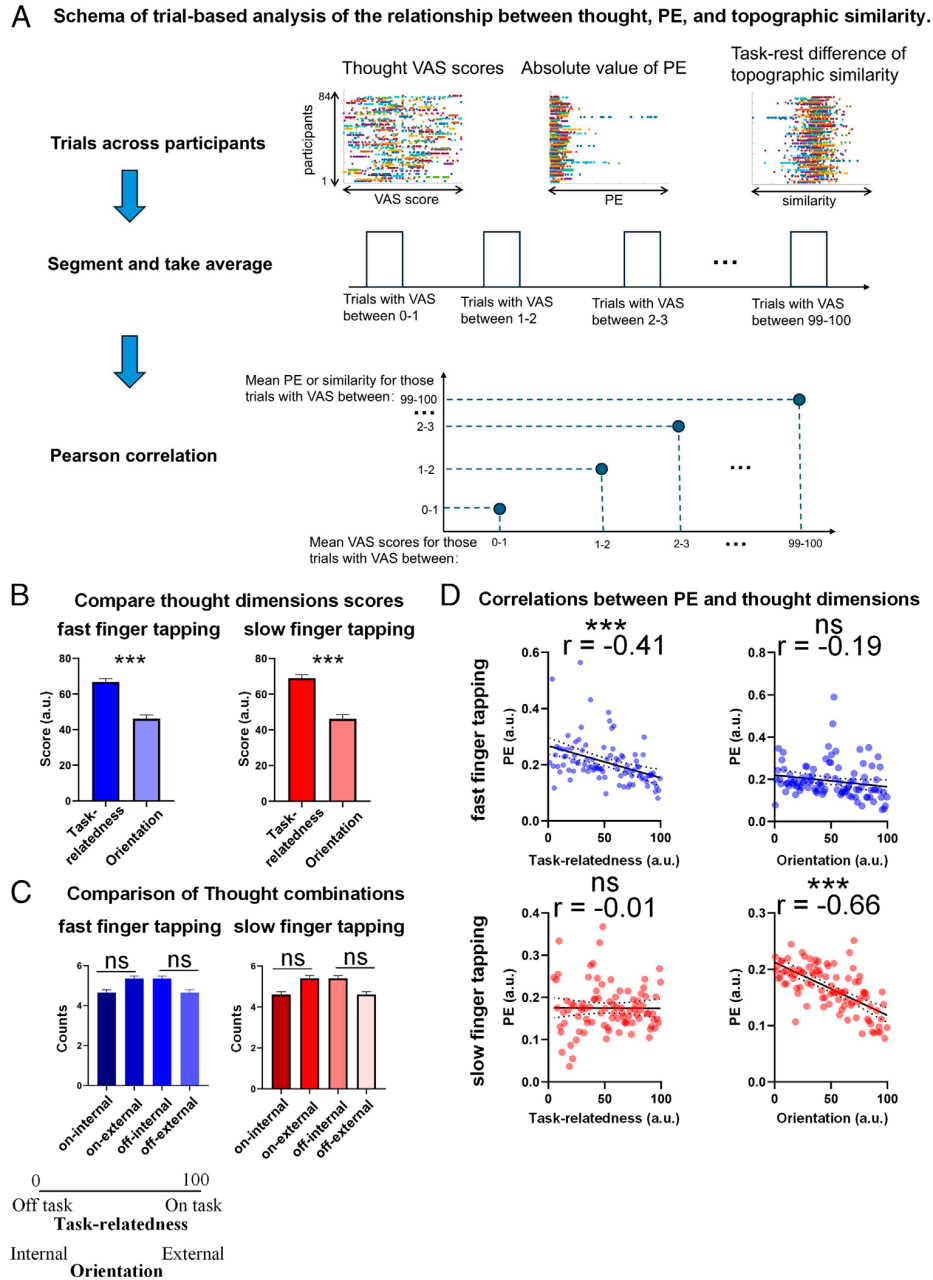
Behavioral results. (*A*) Schema of trial-based analysis of the relationship between thought, PE, and topographic similarity. Data from all participants were aggregated to assess correlations between thought VAS scores and timescale indices at behavioral (PE) and neural (topographic similarity) levels. The analysis was motivated by our focus on the single trial differences between the thought probes themselves as distinct from the intersubject differences related to the different thoughts which is also reflected in and measured by the continuous assessment in our VAS with a grading between the extremes of internal–external and on–off (as distinct from the standardly used binary assessment of internal–external and on–off thoughts). Trial values were averaged within VAS score segments (e.g., 0 to 1, 1 to 2, etc.) to mitigate noise, balance segment weighting, and reduce oversignificance. Pearson correlation analysis was performed on z-scored values (−3 to 3), and absolute PE values were used to provide a stable metric for thought dimensions. (*B*) Comparison between task relatedness and orientation VAS scores. Task relatedness and orientation VAS scores in fast and slow finger tapping are compared by the paired *t* test. The results show that in both speeds, the participants’ scores for thought orientation (internal–external dimension) are significantly lower than for task-relatedness (off-on task dimension). (*C*) Comparison of thought combinations. The counts of trials with on-task and internal, off-task and internal, on-task and external, and off-task and external for each participant were compared by the Friedman test. Although significant differences were found among the four groups during both fast and slow finger tapping, multiple comparison tests showed no significant pairwise differences between the four categories in either fast or slow finger tapping. (*D*) Correlation between PE and thought dimensions. The interaction of orientation and task relatedness with PE during both slow and fast self-paced finger tapping were tested by applying binned correlation. Each dot represents one VAS score on the Y axis and the averaged PE value across all trials with that VAS score on the X axis. Results show that slow speed finger tapping is only related with orientation but not with task-relatedness. While fast finger tapping PE is only related with task-relatedness but not with orientation. Note: Error bars represent SEM; VAS: visual analog scale; PE: precision error; **P < 0.01; ***P < 0.001; ***P < 0.001; ns: none significance.

The PE was used in this study to measure the performance of finger tapping (30, 32, 33). PE captures the deviation between the mean of real ITI and the SI by using the ratio between them: $PE=1-\frac{\mathrm{ITI}}{\mathrm{SI}}$; The closer the PE is to zero, the more it indicates a precise replication of the given SI (Fig. 1*C*). Prior to computing the correlation between thought VAS scores and PE, we calculated the absolute value of PE to provide a more stable metric for assessing the relationship with thought dimensions (Fig. 2*A*).

### EEG Recording and Preprocessing.

EEG was recorded using a 64-channel BrainAmp system (Brain Products) at a sampling rate of 500 Hz. Oz served as the online reference; impedance was kept <5 kÎ©. Data were processed in EEGLAB (34): rereferenced to the average, filtered (1 to 50 Hz), and cleaned using ICA. Bad channels were removed using clean_rawdata, and artifacts (e.g., blinks, saccades) were automatically excluded via MARA plug-in in EEGLAB (35, 36).

### EEG Analysis.

#### Topographic similarity.

**Fig. 3.**
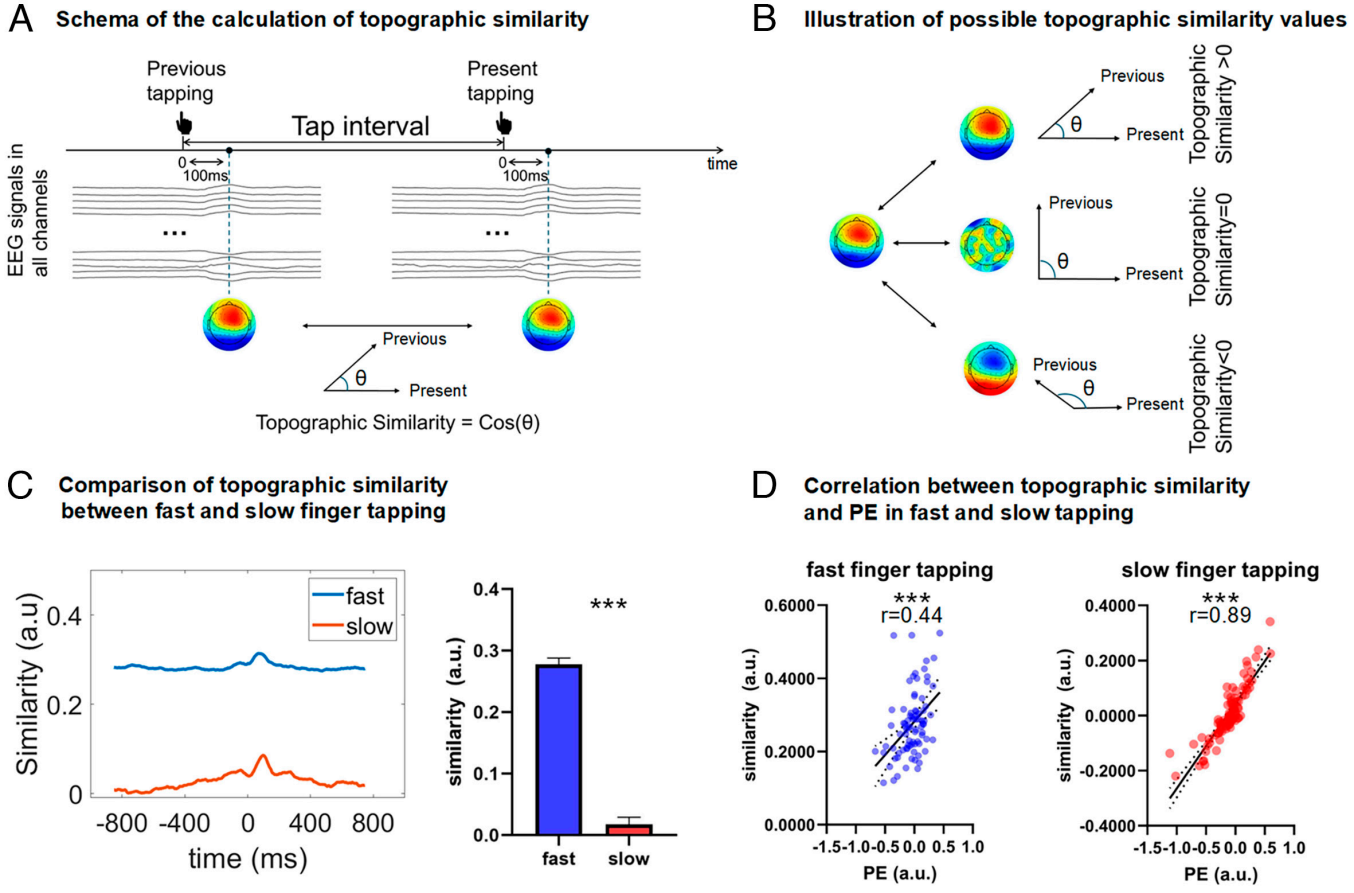
Topographic similarity. (*A*) schema of the calculation of topographic similarity. For each finger tap event, we calculated the topographic similarity between the topographic maps at each time point within the −1,200 ms to 1,200 ms window and the topographic map at the corresponding time point of the previous finger tap event. Topographic similarity was measured by calculating cosine similarity: $\mathrm{Topographic}\mathrm{similarity}=\frac{V_{n-1}\cdot V_{n}}{\left|\left|V_{n-1}\right|\right|\times \left|\left|V_{n}\right|\right|}$, in which $V_{n-1}$ represents the vector of the previous tapping topography, and $V_{n}$ represents the vector of the present tapping topography. (*B*) Illustration of possible topographic similarity values. If the topographic similarity is positive, it indicates a positive interaction between the past and present. If the topographic similarity is close to zero, it suggests a low past–present interaction. If the topographic similarity is negative, it means the current topography has an opposite pattern to the previous one, reflecting a negative interaction between the past and present. (*C*) comparison of topographic similarity between fast and slow finger tapping. The averaged values of topographic similarity between 0 to 300 ms window were used as the peak value. The peak values within each participant were averaged and were compared between fast and slow finger tapping conditions using the paired sample *t* test. The results showed that the topographic similarity in fast finger tapping was significantly higher than in slow finger tapping, suggesting that the impact of previous tapping on the present tapping decreases with longer intervals. (*D*) Correlation between topographic similarity and PE in fast and slow tapping. The correlation between the peak values of topographic similarity and the PE in both slow and fast tapping conditions were examined by Pearson correlation. The results indicated a significant correlation between PE and topographic similarity at both speeds, suggesting that topographic similarity is related to PE regardless of the interval length. Note: Error bars represent SEM; PE: precision error; ***P < 0.001.

To assess temporal integration, EEG data were segmented into −1,200 to 1,200 ms epochs around each tap. For each time point, topographic maps were computed using a ±50 ms window and compared with maps from the preceding tap using cosine similarity (Fig. 3*A*). Positive values indicated similar spatial patterns, while negative or near-zero values reflected dissimilarity (Fig. 3*B*). Baseline topographic similarity was computed from resting-state EEG using the same intervals (0.8 s for fast, 1.9 s for slow) and subtracted from task-based values to isolate task-specific effects (*SI Appendix,* Fig. S1 *A* and *B*).

#### Phase locking value and phase shuffling.

To examine whether topographic similarity was driven by phase coherence, we computed phase locking value (PLV) between adjacent tapping events using the Hilbert transform. PLV was averaged across electrodes to yield a global phase coherence index. To test phase specificity, phase-shuffled surrogate EEG signals (preserving spectral power but disrupting phase) were generated. PLV and topographic similarity were recalculated on the shuffled data. Correlations between topographic similarity with PE and thought ratings were calculated on shuffled data to assess phase-based contributions.

#### Event-related spectral perturbation.

As a control, we analyzed ERSP at Fz, Cz, and Pz. Epochs were time-locked to taps (−800 to 800 ms) and baseline-corrected (−800 to −100 ms). Cluster-based permutation tests (1,000 iterations) identified time–frequency differences (1 to 40 Hz) between conditions (|t| > 2.0), and correlations with PE and thought ratings (|r| > 0.2).

### Statistical Analysis.

#### Trial-based correlation.

Trial data from all participants were pooled, and Pearson correlations were computed between z-scored VAS ratings (−3 to 3) and trial-level measures. To reduce noise and balance segment representation, trials were binned by VAS scores (e.g., 0 to 1, 1 to 2…). This approach emphasizes within-trial variability in cognitive state rather than between-subject traits. The use of continuous VASs allowed for finer-grained assessments than traditional binary probes, as reflected by unimodal score distributions across participants (*SI Appendix,* Fig. S2). Analyses were performed separately for PE and topographic similarity to capture distinct relationships with thought content.

#### Calculation of the thought’s impact on topographic similarity.

To isolate task-specific effects, we computed topographic similarity during resting state at the same intervals (0.8 s or 1.9 s) and subtracted it from task-based values (pseudotrial method). The resulting task–rest differences were correlated with thought ratings, and the absolute correlation coefficient was used as a measure of thought’s impact (*SI Appendix,* Fig. S1*B*). To control for tapping interval confounds, we conducted partial correlations using ITI–SI differences as covariates.

#### Comparing thought impact on topographic similarity between thought dimensions.

**Fig. 4.**
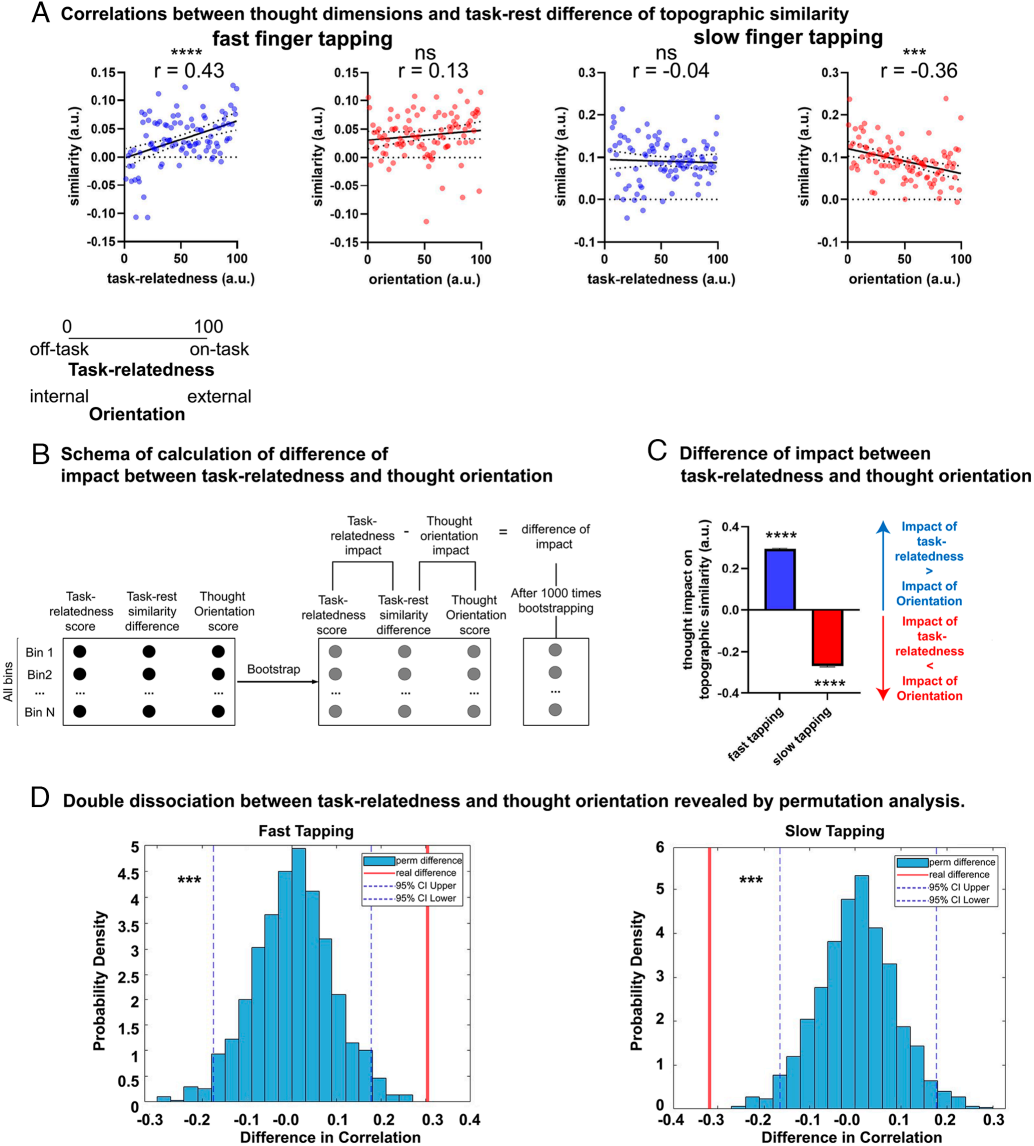
Thought dimension impact on topographic similarity. (*A*) Correlations between thought dimensions and task-rest difference of topographic similarity. The results show that for fast finger tapping, task-rest difference of topographic similarity is correlated with task-relatedness but not thought orientation. Whereas for slow finger tapping, task-rest difference of topographic similarity correlates only with thought orientation but not with task-relatedness. These results reveal a double dissociation between the neural basis of the task-relatedness dimension and the thought orientation dimension with respect to fast vs. slow finger tapping. (*B*) Schema of calculation of difference of impact between task-relatedness and thought orientation. We performed 1,000 bootstrap resamples for the segmented data, calculating the impact of thought on topographic similarity for both thought dimensions and then computed the difference between task-relatedness and thought orientation. A one-sample *t* test was used to compare the distribution of these 1,000 differences to 0, indicating whether task-relatedness or thought orientation contributed more to the topographic similarity during slow and fast finger tapping. (*C*) Difference in the impact between task-relatedness and thought orientation. We observed a double dissociation between the two thought dimensions. Specifically, during fast finger tapping, the impact of task-relatedness on topographic similarity was significantly greater than that of thought orientation. Conversely, during slow finger tapping, thought orientation shows a stronger impact than task-relatedness on topographic similarity. (*D*) Double dissociation between task relatedness and orientation revealed by permutation analysis. During fast finger tapping, topographic similarity was more strongly influenced by task-relatedness than by thought orientation. In contrast, during slow finger tapping, thought orientation exerted a greater influence on topographic similarity than task-relatedness. The red solid line represents the observed difference, while the blue dashed lines indicate the upper and lower edges of the 95% CI of the permutation distribution. Note: Error bars represent SEM; SI: standard interval; PE: precision error; ***P < 0.001.

We assessed which thought dimension more strongly influenced topographic similarity by computing the difference between their effects across 1,000 bootstrap resamples. A one-sample *t* test was used to test whether the mean difference deviated significantly from zero. Additionally, we conducted a 1,000-iteration permutation test on this difference to evaluate whether the observed effect exceeded chance (37). Significant positive or negative results indicated stronger contributions from task-relatedness or orientation, respectively (Fig. 4*C*).

#### Linear Mixed Models and GAMs.

To account for interindividual variability and examine nonlinear effects, we used linear mixed-effects models (LMMs) and GAMs. Trials >3 SDs from the mean were excluded, and remaining data were binned by thought ratings within each participant. LMMs were used to assess associations between thought dimensions and outcome variables (PE or topographic similarity), while GAMs captured potential nonlinear trends. Residuals from GAMs were reentered into LMMs to isolate linear effects. To test whether nonlinearity differed across tapping conditions, we fit interaction GAMs with condition-specific smooth terms using the mgcv package in R (38).

#### Bayesian Nonlinear Mediation Analysis Using Smooth Functions of Thought Variables.

We further examined whether nonlinear components of thought mediated their effects on behavior and brain activity using a Bayesian mediation framework. A two-step procedure was applied: 1) GAMs estimated smooth terms for each thought dimension; 2) these were entered as mediators in Bayesian mediation models predicting PE or topographic similarity. Parameters were estimated via MCMC sampling, with inference based on 90% credible intervals and posterior direction probabilities. Effects were considered significant if their credible intervals excluded zero.

## Results

### Cognitive Level: Thought Orientation and Task-Relatedness Dimensions Are Dissociable.

To compare the two thought dimensions at the cognitive level, we first compared their two visual analogue scales (VAS) ratings with each other during all conditions (resting state and the two finger tapping tasks). The results show that the participants’ scores for thought orientation are significantly lower than those for task-relatedness in all three conditions [resting state: t(83) = 14.30, *P* < 0.001; fast finger tapping: t(83) = 8.56, *P* < 0.001; slow finger tapping: t(83) = 8.40, *P* < 0.001, Fig. 2*B*, *SI Appendix,* Fig. S4*A*]. Hence, the balance of thought orientation is rated subjectively different than that of task-relatedness. At the subjective level, if participants considered these two dimensions to be two constructs that assess the same underlying thought dimension, we would expect the rating to be similar. Specifically, the VAS scores task-relatedness were around 70 while the scores of thought orientation were around 40 to 50, consistently, in the three conditions (resting state: task-relatedness: mean = 72.75, std = 16.14; orientation: mean = 39.32, std = 15.75; fast tapping: task-relatedness: mean = 66.67, std = 18.04; orientation: mean = 46.10, std = 19.68; slow tapping: task-relatedness: mean = 68.95, std = 17.82; orientation: mean = 46.16, std = 21.18). This pattern of scores indicates that the balance between off vs. off tasks thought leans more toward the on-task end of the spectrum, whereas the internal vs. external balance is more intermediate (i.e., neither one, nor the other).

Next, we raised the question of whether the ratings for the two thought dimensions were correlated to each other. The results showed a significant correlation between the task-relatedness and thoughts orientation dimension scores during the fast finger tapping task [r(82) = 0.32, *P* = 0.003; *SI Appendix,* Fig. S3], whereas no significant correlations were obtained in both resting state and slow finger tapping [resting state: r(82) = 0.10, *P* = 0.377; slow finger tapping: r(82) = 0.20, *P* = 0.075; *SI Appendix,* Fig. S3]. These data suggest that the relation between the two thought dimensions differs across the three conditions with only the fast finger tapping suggesting that they are related to one another, while they remain independent in both rest and slow finger tapping.

Are task-relatedness and thoughts orientation different thought dimensions rather than just different metrics of the same underlying continuum? If they are just different degrees on the same continuum, one would expect high ratings for internal thought to correlate with high ratings for off-task thoughts, while high ratings for external thought should align with high ratings for on-task thoughts. To test for this, we performed a median split on each participant’s thought probes based on their VAS scores. The top 10 trials with the highest scores were classified as on-task or external thought trials, while the lowest 10 trials were classified as off-task and internal thought trials. We then calculated, for each participant, the number of trials that fell into the following four combinations: 1) on-task and internal, 2) off-task and internal, 3) on-task and external, and, 4) off-task and external. Since the counts of the four combinations are not continuous variables, we used the nonparametric Friedman test to determine whether there are significant differences among the counts of the four combinations. This approach revealed no significant differences in these four combinations during the resting state (Friedman statistic = 0.00, *P* > 0.999; *SI Appendix,* Fig. S4*B*). While significant differences were found among the four groups during both fast and slow finger tapping (fast: Friedman statistic = 13.34, *P* = 0.004; slow: Friedman statistic = 17.75, *P* = 0.001), multiple comparison tests showed no significant pairwise differences between the four categories in either fast or slow finger tapping (all *P* > 0.05; Fig. 2*C*; see *SI Appendix,* Tables S1 and Table S2 for detailed results).

Together, these results suggest that while there is some degree of correlation between task-relatedness and thought orientation, there is no strict correspondence between on-task and external thoughts nor between off-task and internal thoughts. This supports the assumption that task-relatedness and thought orientation are indeed distinct dimensions of thought, rather than being different degrees or scales of one and the same dimension. In summary, all three findings, VAS rating degrees, correlation analysis, and association of thoughts, suggest that, based on their subjective assessment, task-relatedness and thought orientation are distinct dimensions rather than variations of one and the same underlying dimension.

### Behavioral Level: Thoughts Orientation Relates to the Longer Timescale of Slow Finger Tapping While Task-Relatedness Relates to the Shorter Timescale of Fast Finger Tapping.

We next investigated the timescales of orientation and task-relatedness. We first observed that PE was not significantly different from 0 in both fast and slow tapping, as indicated by one-sample *t* test [fast: mean (95% CI) = −0.02 (−0.08, 0.04), SD = 0.26, SEM = 0.03, t(83) = 0.71, p_fdr(1) =_ 1.000; slow: mean (95% CI) = 0.04 (−0.01, 0.08), SD = 0.22, SEM = 0.02, t(83) = 1.46, p_fdr(1) =_ 1.000]. These results indicate that the deviation of the participants’ actual tapping intervals from the SI during the experiment was minimal, with no systematic bias observed in either slow or fast finger tapping conditions. We also observed that the PE is not significantly different between fast and slow finger tapping [t(83) = 1.78, *P* = 0.079; *SI Appendix,* Fig. S5]. Moreover, the absolute value of PE is also not significantly different between fast and slow finger tapping [t(83) = 0.12, *P* = 0.904; *SI Appendix,* Fig. S5]. These results suggest that no significant change of PE occurred due to the duration of the tapping intervals, which are longer in slow finger tapping, and shorter in fast finger tapping.

Next, we investigated the interaction of PE with our two thought dimensions during both slow and fast finger tapping. The results show that the PE during slow finger tapping is only related with orientation but not with task-relatedness [task-relatedness: r(90) = −0.01, p_fdr(1) =_ 1.000; orientation: r(93) = −0.66, p_fdr(1)_ < 0.001; Fig. 2*D*]. While fast finger tapping PE only relates with task-relatedness thought but not with orientation [task-relatedness: r(95) = −0.41, p_fdr(1)_ < 0.001; orientation: r(98) = −0.19, p_fdr(1) =_ 0.072; Fig. 2*D*]. Together, these results show double dissociation between timescales (slow and fast finger tapping) and thought dimensions (task-relatedness, thought orientation): Thought orientation is associated with longer timescales of the slow finger tapping, whereas task-relatedness relates to shorter timescale of the fast finger tapping.

### Behavioral Level: Task-Relatedness Modulates PE in a Nonlinear Manner While Orientation Modulates PE in Linear Way.

To control for individual variability, we first applied LMMs and observed a marginal negative association between task-relatedness and PE in the fast tapping condition [Beta = −0.0006, t = −1.87, 95% CI (−0.001, 0.000)], whereas orientation showed no effect [Beta = 0.000, t = 0.04, 95% CI (−0.0005, 0.0005)]. To test for potential nonlinear structure, we fitted GAMs and found that task-relatedness exhibited mild nonlinearity (edf = 2.23, *P* = 0.10), which accounted for its original effect; after regressing out this component, the LMM residuals showed no significant relationship [Beta = 0.0001, t = 0.25, 95% CI (−0.0005, 0.0007)]. Conversely, orientation revealed a strong linear association after adjustment [Beta = 0.0010, t = 3.83, 95% CI (0.0005, 0.0015)], with GAM confirming a strictly linear structure (edf = 1.00, *P* = 0.0002). In the slow tapping condition, task-relatedness again showed a significant negative effect in the initial LMM [Beta = −0.0010, t = −2.85, 95% CI (−0.0017, −0.0003)], but was later explained by pronounced nonlinearity in the GAM model (edf = 5.03, *P* = 0.0009); controlling for this, the effect was eliminated [Beta = 0.0000, t = −0.02, 95% CI (−0.0007, 0.0007)]. Orientation remained a robust linear predictor (edf = 1.04, *P* < 0.0001), demonstrating consistency across timescales. Full model details are provided in *SI Appendix,* Table S3, including effect sizes, SE, *t* values, and 95% CI for the LMMs, as well as estimated degrees of freedom (edf), F-values, and *P*-values for the GAMs.

To formally assess whether nonlinear processes mediated these effects, we conducted Bayesian mediation analyses. Task-relatedness showed significant indirect effects in both fast and slow conditions [fast: mean = −0.11, 90% CI (−0.20, −0.04); slow: mean = −0.12, 90% CI (−0.16, −0.08)], while direct effects were nonsignificant. This indicates a suppression mechanism where the nonlinear mediator reversed the direct trend. Orientation, by contrast, showed no significant indirect or direct effects but a consistently significant total effect [e.g., fast: mean = −0.18, 90% CI (−0.25, −0.10)], supporting a direct, linear contribution.

Finally, to compare the magnitude of nonlinear modulation across temporal contexts, we applied an interaction GAM. Task-relatedness showed significantly stronger nonlinearity in the slow vs. fast tapping condition (F = 2.45, *P* = 0.027), whereas the linear structure for orientation was stable across conditions (F = 0.64, *P* = 0.493).

Taken together, these results indicate that the nonlinear relationship between task-relatedness and PE suppresses its overall effect, with this suppression being more pronounced under the slow tapping condition. In contrast, orientation contributes to PE in a consistent and robust linear fashion, and this linear effect may become more detectable when the influence of task-relatedness is masked by nonlinearity. Full model details and diagnostics are presented in *SI Appendix,* Tables S4–S6.

### Neural Level: Topographic Similarity Correlates with the PE During Both Slow and Fast Finger Tapping.

We so far showed differences in thought orientation (internal–external dimension) and task-relatedness (off-on task dimension) in both their cognitive evaluation and their interactions with short vs. long behavioral timescales. Next, we investigated whether these timescale/duration differences on the behavioral level also manifested at the neural level. To operationalize duration on the neural level, we used the topographic similarity to measure the degree of similarities in the topographic distribution of the EEG signal of the present time points with the ones in the past (20, 21, 39). Given that task-relatedness and thought orientation were differentially related with the short vs. long timescales for finger tapping at the behavioral level, we expected to observe analogous differences in their underlying neural duration, in terms of topographic similarity.

The results show that the topographic similarity during fast finger tapping was significantly higher than in slow finger tapping [t(83) = 21.01, *P* < 0.001, Fig. 3*C*]; This suggests that the influence of preceding finger taps is stronger during fast tapping, where time intervals are shorter, compared to slow tapping with longer intervals. This suggests that the longer timescale of slower finger tapping and the shorter timescale of faster finger tapping are also manifest at the neural level in a corresponding manner.

Next, we asked whether these distinct neural durations, measured by topographic similarity during fast and slow finger tapping, were related to the PE. For that purpose, we examined the correlation between the peak values of topographic similarity and the PE in both slow and fast tapping conditions by Pearson correlation. The results indicated a significant correlation between PE and topographic similarity at both speeds [fast: r(80) = 0.44, p_fdr(1)_< 0.001; slow: r(80) = 0.89, p_fdr(1)_ < 0.001, Fig. 3*D*]. These findings demonstrate a clear relationship between PE at the behavioral level and the topographic similarity at the neural level.

Finally, to ensure that the relationship between topographic similarity and PE was truly driven by the task itself—namely, the finger tapping and its time intervals—we also calculated the difference in topographic similarity between task and resting states and then examined the correlation between the rest-task difference and the PE of the finger tapping. As in the above results, this again revealed significant correlation of the task-rest differences of the topographic similarity with the PE in both slow and fast finger tapping [fast: r(80) = 0.41, p_fdr(1)_ = 0.001; slow: r(80) = 0.83, p_fdr(1)_ < 0.001, *SI Appendix,* Fig. S1*C*].

### Neural Level: Task-Relatedness and Thought Orientation Relate Differently to Topographic Similarity During Fast and Slow Finger Tapping.

Are the different timescales at the neural level during slow and fast finger tapping, as measured by the topographic similarity, related to our two thought dimensions, namely task-relatedness and thought orientation? To address this, we calculated the task-rest difference of topographic similarity by subtracting the resting state topographic similarity (used as a baseline) from the corresponding similarity at each timescale during the finger-tapping task (0.8 s in resting state corresponds to fast tapping, 1.9 s in resting state corresponds to slow tapping). This approach isolated the true effect of thoughts on topographic similarity during the finger tapping task itself by removing resting-state influences that are carried over to the task state. Following the approach used in the behavioral analysis, we calculated the single-trial based correlation between the task-rest difference of topographic similarity and the thought dimensions. The results show that for fast finger tapping, task-rest difference of topographic similarity is correlated with task-relatedness [r(95) = 0.43, p_fdr(3)_ < 0.001] but not thought orientation [r(98) = 0.13, p_fdr(3) =_ 1.000, Fig. 4*A*]. Whereas for slow finger tapping, the task-rest difference of topographic similarity correlates only with thought orientation [r(91) = −0.36, p_fdr(3)_ < 0.001] but not with task-relatedness [r(91) = −0.04, p_fdr(3) =_ 1.000]. These results reveal a double dissociation between the neural basis of the task-relatedness dimension and the thought orientation dimension with respect to fast vs. slow finger tapping.

As another control, we tested whether topographic similarity across SI during the resting state were related to thought. The results show that in both fast and slow conditions (with SI = 0.8 s and SI = 1.9 s, respectively), resting-state topographic similarity was not correlated with either thought dimension. Specifically, for fast SI: task-relatedness [r(87) = 0.03, p_fdr(3)_ = 1.000] and orientation [r(98) = −0.13, p_fdr(3)_ = 1.000]; and for slow SI: task-relatedness (r(92) = 0.24, p_fdr(3)_ = 1.000) and orientation [r(98) = 0.07, p_fdr(3)_ = 1.000] showed no significant correlations (*SI Appendix,* Fig. S6). These results suggest that the interaction between thought and PE is specifically related to the actual tapping and the participants’ PE rather than the SI themselves.

In a second step, we performed 1,000 bootstrap resamples across all trials for all participants, calculating the correlation between each thought dimension and task-rest difference of topographic similarity and then computed the difference between the task-relatedness and the thought orientation dimensions. A one-sample *t* test was used to compare the distribution of these 1,000 differences to 0, indicating whether task-relatedness or thought orientation contributed more to the topographic similarity (Fig. 4*B*). These results demonstrate a double dissociation between the two thought dimensions. Specifically, during fast finger tapping, the impact of task-relatedness on topographic similarity was significantly greater than thought orientation [mean = 0.29, SD = 0.12, SEM = 0.004, t(98) = 74.47, *P* < 0.001; Fig. 4*C*]. Conversely, during slow finger tapping, the impact of thought orientation on topographic similarity was significantly greater than that for task-relatedness [mean = −0.27, SD = 0.13, SEM = 0.004, t(98) = 65.20, *P* < 0.001; Fig. 4*C*]. Furthermore, the permutation results also show the same double dissociation between task relatedness and orientation [fast tapping: observed difference = 0.304, permutation difference: mean (95%CI) = 0.002 (−0.176, 0.177), p_fdr_ < 0.001; slow tapping: observed difference = −0.319, permutation difference: mean (95%CI) =0.003 (−0.164, 0.178), p_fdr_<0.001; Fig. 4*D*].

To confirm that the observed double dissociation of thought dimension on topographic similarity was due to the influence of the thoughts themselves, rather than differences in motor tapping behavior, we included the difference between ITI and SI as a covariate and conduct partial correlations. The results showed that even after controlling for the covariate, the effect of task-relatedness thought during fast finger tapping remained significant [r(94) = 0.43, *P* <0.001] while thought orientation was not [r(94) = 0.14, *P* = 0.159]. Conversely, for slow finger tapping, task-relatedness was not significant [r(93) = −0.02, *P* = 0.883], while thought orientation remained significant [r(93) = −0.36, *P* < 0.001].

Together these results indicate that, after controlling the impact from the tapping interval in fast finger tapping, task-relatedness significantly influences the topographic similarity, while thought orientation does not. In contrast, the situation is reversed during slow finger tapping, whereby thought orientation significantly influences topographic similarity whereas task-relatedness does not. Since we also included finger tapping behavioral performance variables as covariates, these correlations strongly suggest that the double dissociation of task-relatedness and thought orientation for the topographic similarity at long vs. short timescales is driven by the thoughts themselves and their different timescales, rather than by the tapping behavior per se.

### Neural Level: Nonlinear Contributions to the Double Dissociation Between Thought Dimensions and Topographic Similarity.

Initial linear regression revealed a double dissociation: Task-relatedness correlated with topographic similarity under fast tapping, while orientation was associated under slow tapping. To test for nonlinear contributions, we applied LMMs and GAMs. In the fast condition, the task-relatedness effect in the LMM [Beta = 0.0004, t = 2.57, 95% CI (0.0001, 0.0008)] disappeared after removing nonlinear components [Beta = 0.0000, t = −0.24, 95% CI (−0.0004, 0.0003)], with GAM confirming strong nonlinearity (edf = 3.74, *P* = 0.001). Orientation showed a weak, nearly linear pattern (edf = 1.00, *P* = 0.04). In the slow condition, though not significant, task-relatedness remained linear (edf = 1.00, *P* = 0.16), while orientation showed nonlinearity (edf = 3.15, *P* = 0.37) (see *SI Appendix,* Table S7 for the detailed results, including effect size, SE, and 95%).

Bayesian mediation analyses further confirmed this dissociation. Under fast tapping, task-relatedness showed a significant indirect effect on topographic similarity [mean = 0.14, 90% CI (0.08, 0.20)], with no direct effect, indicating a nonlinear mediation pathway. Orientation showed no credible mediation despite a large numerical estimate. Under slow tapping, only orientation yielded a small but credible indirect effect [mean = 0.01, CI (0.01, 0.02)]; task-relatedness effects were nonsignificant and unstable (see *SI Appendix,* Table S8 for the mediation results). Model convergence issues in the slow condition for task-relatedness (rÌ‚ > 1.1) were traced to perfect collinearity (r = 1.00) between the predictor and its smooth term, implying an essentially linear relationship (*SI Appendix,* Table S9).

Together, these results demonstrate a nonlinear double dissociation: Task-relatedness nonlinearly predicts topographic similarity only at short timescales, while orientation does so only at long timescales.

### ERSP Results: Beta–Gamma Power Tracks Behavioral Precision in Fast Tapping but Not Thought.

To examine time-frequency dynamics, we conducted cluster-based permutation tests comparing fast and slow tapping conditions at Fz, Cz, and Pz (1,000 permutations, cluster threshold |t| > 2.0). Each electrode revealed four significant clusters (*P* < 0.05, corrected), primarily within the beta/gamma (13 to 40 Hz) and theta (3 to 8 Hz) ranges. Fast tapping elicited greater high-frequency power both before (−800 to 0 ms) and after tapping (230 to 798 ms), while theta-band differences spanned pre- and early postmovement windows.

We then assessed correlations between ERSP power and PE using cluster-based permutation analysis (r > 0.2, 1,000 permutations). Under the fast tapping condition, significant clusters were found at all electrodes—especially at Fz (e.g., 3 to 32.4 Hz, −800 to −450 ms; 10.6 to 39.2 Hz, 202 to 798 ms; *P* < 0.001). Cz and Pz also showed multiple beta-range clusters in the similar time and frequency periods. In contrast, the slow condition yielded no significant clusters at any electrode, suggesting that the association between ERSP power and PE is specific to the fast tapping condition, particularly within high-frequency bands around movement execution.

In contrast, no significant ERSP–thought correlations were observed at any electrode or condition, indicating that oscillatory power does not reliably track subjective thought dimensions in this context. All ERSP results are shown in *SI Appendix,* Figs. S7-S9

### Thought Is Modulated by Phase Dynamics Rather Than Power Changes.

To elucidate the neural mechanisms underlying thought, we investigated whether its influence on neural oscillatory activity is mediated by phase dynamics rather than power fluctuations. This inquiry was prompted by the absence of robust associations between thought and conventional ERSP markers.

**Fig. 5.**
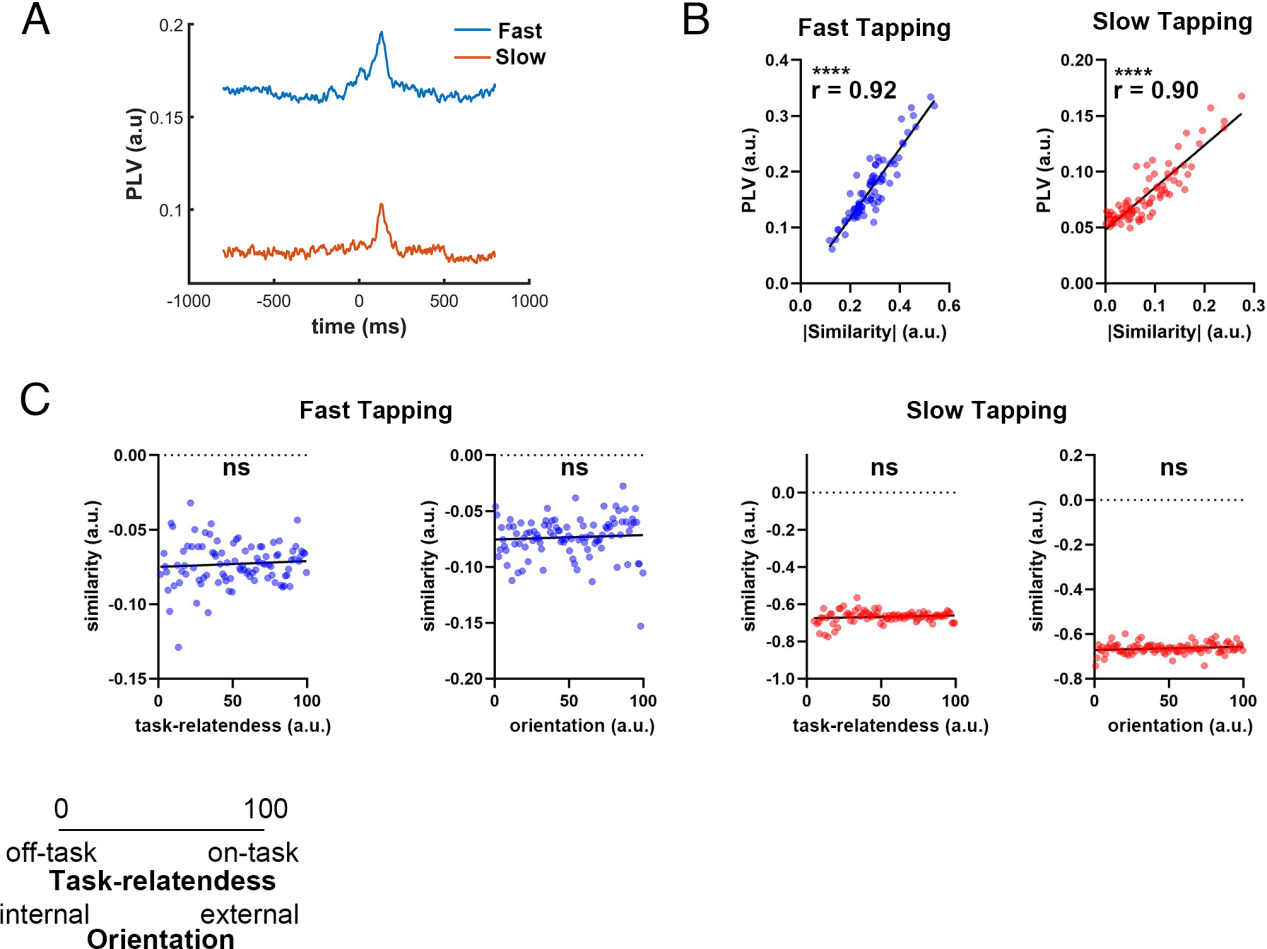
Phase-based neural dynamics underlie topographic similarity and its association with thought. (*A*) Grand-averaged PLV across trials and electrodes under fast and slow tapping conditions. A distinct PLV peak emerged within the 0 to 300 ms poststimulus window in both conditions, aligning with the topographic similarity peak. (*B*) Scatterplots showing the strong positive correlation between mean PLV (0 to 300 ms) and absolute topographic similarity across participants, indicating that topographic similarity is grounded in phase synchrony. (*C*) Correlation plots between topographic similarity and thought probe ratings (task-relatedness and orientation) under fast and slow tapping after phase shuffling. The original correlations disappeared following phase randomization, suggesting that phase dynamics are essential to the link between neural activity and thought.

To investigate phase-based mechanisms, we computed the phase locking value (PLV) between finger taps. A PLV peak within 0 to 300 ms posttap was observed under both tapping conditions (Fig. 5*A*), mirroring the topographic similarity profile. Mean PLV in this window strongly correlated with absolute topographic similarity [fast: r(80) = 0.92; slow: r(80) = 0.90; both *P* < 0.0001; Fig. 5*B*]. Because topographic similarity includes negative values (especially in slow tapping, *SI Appendix,* Fig. S10*A*), we used its absolute values and excluded ±3 SD outliers to avoid piecewise distortions. These findings suggest that topographic similarity reflects a phase-based, not amplitude-driven.

To validate this, we performed phase-shuffling. Both the 0 to 300 ms PLV and topographic similarity peaks disappeared (*SI Appendix,* Fig. S10 *B* and *C*), and their correlations with PE were abolished [fast tapping: r(80) = −0.02, *P* = 0.880; slow tapping: r(80) = −0.03, *P* = 0.745; *SI Appendix,* Fig. S10*D*]. Likewise, all thought–similarity correlations became nonsignificant [all p_fdr(3)_ = 1.00; task-relatedness during fast tapping: r(94) = 0.16; orientation during fast tapping: r(98) = 0.06; task-relatedness during slow tapping: r(91) = 0.14; orientation during slow tapping: r(98) = 0.17; Fig. 5*C*], confirming that intact phase structure is necessary for thought-related neural dynamics.

In conclusion, these findings suggest that the relationship between thought and neural oscillatory activity is mediated primarily by phase dynamics rather than power changes.

### Replication of Main Findings in an Independent Dataset.

To evaluate the robustness of our findings, we replicated all major analyses using an independent dataset. Behaviorally, thought orientation consistently scored lower than task-relatedness across all conditions, reaffirming its alignment with longer cognitive timescales. PE patterns replicated the original double dissociation: Task-relatedness predicted PE under fast tapping, while orientation predicted PE under slow tapping. These relationships were further supported by linear mixed models, GAMs, and Bayesian mediation analyses, which again revealed nonlinear suppression effects for task-relatedness and robust linear effects for orientation.

At the neural level, topographic similarity was higher during fast compared to slow tapping and correlated with PE in both conditions. Importantly, the double dissociation between thought dimensions and neural similarity also replicated: Task-relatedness modulated similarity in fast tapping, whereas orientation did so in slow tapping. These effects were characterized by nonlinear interactions and were mediated by distributed phase coherence, as shown by phase-locking value analyses and phase-shuffling controls. Time-frequency analyses (ERSP) further confirmed that beta-band power during fast tapping was selectively associated with PE, replicating findings from the main dataset.

Together, these results reinforce the dissociable timescale mechanisms of task-relatedness and thought orientation across behavior and neural dynamics. Full statistical details and visualizations are available in *SI Appendix*.

## Discussion

By investigating how different dimensions of thought interact with behavioral and neural timescales, we demonstrate that: 1) thought orientation and task-relatedness differ in subjective experience; 2) behaviorally, orientation is associated with longer timescales (slow tapping) and task-relatedness with shorter timescales (fast tapping); 3) neurally, topographic similarity is modulated by orientation over long and by task-relatedness over short timescales; 4) these effects are mediated by distinct nonlinear mechanisms; and 5) are fundamentally phase-based. Together, these findings demonstrate that task-relatedness and thought orientation operate at dissociable behavioral and neural timescales.

### Task-Relatedness and Thought Orientation Operate in Dissociable Timescales at the Behavioral Level.

At the behavioral level, task-relatedness and thought orientation operate on distinct timescales, as measured by self-paced finger tapping. Task-relatedness is associated with PE during fast tapping (short timescale), while thought orientation is related with lower PE during slow tapping (long timescale), revealing a double dissociation. LMM and GAM analyses revealed distinct dynamics underlying these behavioral effects. For task-relatedness, nonlinear trends obscured an initial linear association with PE, especially during slow tapping. In contrast, thought orientation maintained a stable linear association across both tempos, suggesting distinct temporal profiles (see *SI Appendix,* Fig. S20 for an example).

Bayesian mediation analyses supported this dissociation: Task-relatedness showed predominantly indirect, nonlinear associations with PE, while orientation showed primarily direct, linear ones. Under slow tapping, task-relatedness displayed stronger nonlinearity, particularly near ambiguous VAS scores (~50), increasing behavioral variance (*SI Appendix,* Fig. S20). This suggests task-relatedness reflects temporal segregation over shorter durations, while the stable linear pattern for orientation implies more robust temporal integration.

These results point to distinct temporal processing modes for the two thought dimensions: Task-relatedness is linked to short-timescale temporal segregation, while orientation aligns with long-timescale temporal integration. Our behavioral results echoes prior findings showing greater behavioral variability during off-task states (40). The current findings also show that variability (measured by the PE) across short vs. long timescales, is associated with the distinct thought dimensions of task-relatedness and thought orientation. This effect may arise from natural differences in the temporal integration length of these thought dimensions requiring different degrees of temporal integration. Given that thought orientation is related to slow finger tapping, we assume that it requires integration of information over a longer duration, and thus a longer timescale, whereas task-relatedness information is integrated over a shorter timescale.

### Task-Relatedness and Thought Orientation Operate in Dissociable Timescales at the Neural Level.

At the neural level, topographic similarity—quantifying the influence of the past on the present (20)—was associated with PE across both fast and slow tapping. Higher similarity correlated with greater PE, suggesting that tapping precision relates to the duration of ongoing temporal integration across time. Critically, we observed a double dissociation between thought dimensions and neural timescales: Topographic similarity during fast tapping was associated with task-relatedness, while during slow tapping it related to thought orientation. This suggests that task-relatedness engages short-range neural integration, whereas orientation involves longer-range integration, consistent with their distinct temporal profiles.

**Fig. 6.**
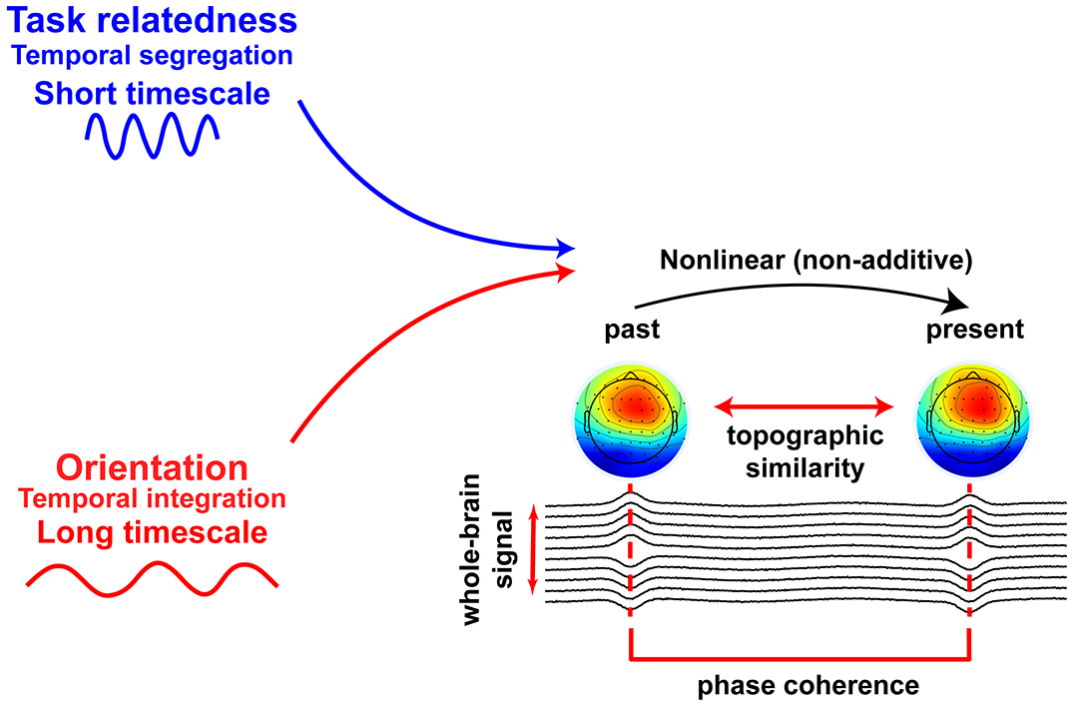
Schematic of the proposed neural mechanism underlying the double dissociation. Task-relatedness is associated with topographic similarity during fast tapping (short timescale), indicating its relationship with temporal segregation. Thought orientation is associated with topographic similarity during slow tapping (long timescale), reflecting its relationship with temporal integration windows. These effects reveal distinct, nonadditive phase-based neural mechanisms for the two thought dimensions across temporal scales.

Neural-level LMM and GAM analyses revealed distinct nonlinear associations: Task-relatedness modulated topographic similarity during fast tapping, while orientation did so during slow tapping. These effects diverged from their behavioral counterparts, likely due to the nonadditive nature of topographic similarity, which captures cross-temporal neural dependencies (24–27). While conventional models assume additive pre–post stimulus effects (1, 2, 12), recent evidence highlights a nonadditive relationship, where prestimulus alpha dynamics modulate trial-to-trial variability (TTV) via TTV quenching (24–27). Our findings align with this view: Using a stimulus-free tapping paradigm, we interpret the nonlinear associations as evidence of cross-temporal integration (Fig. 6).

### Task-Relatedness Is Associated With Temporal Segregation While Thought Orientation Requires Temporal Integration.

The dissociation between task-relatedness and orientation may reflect differences in temporal integration demands (23, 41–43). Fast tapping likely engages rapid updates and short-range segregation of input, supporting task-focused processing (44–46). In contrast, slow tapping allows broader integration over time, drawing on memory to support internally oriented thoughts (44–46). These mechanisms align with our observed timescale effects and support a layered organization of cognitive processing (Fig. 6).

Finally, topographic similarity was closely linked to PLV, and disrupting phase structure eliminated its association with thought. This suggests that the neural correlates of spontaneous thought are fundamentally phase-based. In line with prior work (47, 48), our results support the view that internally directed cognition relies on distributed phase synchrony rather than localized amplitude-based signals (2, 17, 25, 49).

## Limitations

We conducted these analyses on two independent datasets, and the main findings—including the double dissociation between thought dimensions and timescales at both behavioral and neural levels—were fully replicable. In both datasets, the nonlinear mechanisms underlying these dissociations, as revealed by LMMs and GAMs, were also consistently observed. This convergence across analytical approaches and datasets reinforces the robustness and generalizability of our findings. Nonetheless, several limitations should be acknowledged.

First, the gender distribution in the second dataset was not perfectly balanced, which may have introduced potential gender-related sampling bias.

Second, while our findings strongly suggest functional separability between task-relatedness and thought orientation, both dimensions were inferred from VAS ratings, which are moderately correlated. However, the consistent double dissociation observed across behavior and EEG indicates that the two dimensions are associated with distinct processes, despite their subjective correlation.

Third, we did not directly measure thought dynamics. Instead, we inferred them through subjective reports and their interactions with long and short timescales, assessed via finger tapping and topographic similarity. While this is a common challenge in the field of thought and consciousness research, where direct measurement is generally not feasible, we acknowledge that causal evidence (e.g., via experimental manipulation or intervention) would further strengthen our claims. However, the converging evidence from two independent datasets already provides compelling support for the underlying distinction. In addition, we employed Bayesian mediation analyses to examine whether the nonlinear mechanisms served as intermediaries in the relationship between thought and both PE and topographic similarity. While we are fully aware that mediation models do not establish true causality, they can nonetheless offer valuable insights into potential causal pathways and help uncover the latent structure of these thought–timescales (behavioral and neural) associations.

Fourth, our study focused on a single task—finger tapping—which may raise concerns about generalizability to other cognitive domains. That said, finger tapping is particularly well-suited for probing timescale-dependent dynamics due to its continuous and temporally structured nature (11, 17, 18, 50). In future studies, we plan to incorporate a broader range of experimental paradigms—such as the Sustained Attention to Response Task (SART), go/no-go tasks, and simple reaction time paradigms—to further test the generalizability of our findings. Specifically, we aim to examine whether the distinct timescales associated with different dimensions of thought can be consistently observed across diverse cognitive tasks. This approach will substantially extend the scope of the present work and strengthen the theoretical validity of our framework.

Finally, given that thought evolves continuously over time (4, 5), if different dimensions of thought operate on distinct timescales, there should theoretically exist a transition point or intersection between them. However, as distinguishing thought dimensions based on their temporal characteristics is a relatively novel approach, the present study focused on two thought dimensions—task-relatedness and thought orientation—that we hypothesized to differ substantially in timescale, and tested them using two discrete tapping speeds (0.8 s and 1.9 s). While our findings support a dissociation, with task-relatedness associated with shorter timescales and orientation with slower ones, we acknowledge that using only two tapping speeds limits the granularity with which we can capture the transitional dynamics between thought states. In future work, we plan to address this by employing paradigms that allow for continuous variation in tapping speed, enabling a more fine-grained characterization of temporal transitions between distinct cognitive states.

Together, these limitations do not undermine the core conclusions of our study, but rather point to important directions for future research.

## Conclusion

Altogether, we demonstrate a double dissociation of two spontaneous thought dimensions in their timescale at both behavioral and neural levels. Task-relatedness operates at a shorter timescale, whereas thought orientation relies on a longer timescale. This supports the conclusion that task-relatedness (on vs. off-task) and thought orientation (internal vs. external) are indeed distinct dimensions of spontaneous thought, which are processed along different durations (short vs. long) thus reflecting distinct timescales.

Overall, these findings offer valuable insights into how our spontaneous thought processes are modulated by and interact with different timescales and thus entail varying temporal frameworks. This deepens our understanding of the temporal structure and temporal dynamics of thought; that is, thought dynamics (4). This temporal and ultimately spatiotemporal approach to understanding thought is consistent with the recently introduced novel umbrella framework of “Spatiotemporal Neuroscience” which, in a nutshell, focuses on the temporal-dynamic and spatial-topographic shaping of cognitive functions like thought and others (6, 7, 51, 52).

## Supplementary Material

Appendix 01 (PDF)

## Data Availability

Anonymized EEG and behavioral data have been deposited in the online repository Distinct timescales dissociate spontaneous thought dimensions (https://osf.io/ndcxr/) (53).
