# Supplementary material for: Distinct timescales dissociate spontaneous thought dimensions
Source: Proc Natl Acad Sci U S A. 2025 Sep 17;122(38):e2427088122. doi: 10.1073/pnas.2427088122 (PMC12478187; doi:10.1073/pnas.2427088122)
Supplement: Supplementary file 1 — Appendix 01 (PDF) [file pnas.2427088122.sapp.pdf]

Supplements

Method details

Participants

Dataset 1. Eighty-six right-handed adults participated in the study (41 female; age range = 18-30 years; mean age = 21 years, SD = 2.3 years). All had normal or corrected-to-normal vision and reported no neurological or psychiatric conditions that might affect performance. Of these, 2 were excluded as the EEG data were of sufficient quality. Ultimately, 84 participants' data were included in the final analyses. The experimental protocols were approved by the research ethics committee of the Shenzhen University and the University of Ottawa. Written informed consent was obtained from each participant prior to study participation.

Dataset 2. Thirty-five right-handed adults participated in the study (11 female; age range = 18-28 years; mean age = 22.6 years, SD = 2.4 years). All had normal or corrected-to-normal vision and reported no neurological or psychiatric conditions that might affect performance. The experimental protocols were approved by the research ethics committee of the University of Ottawa and Zhejiang University. Written informed consent was obtained from each participant prior to study participation.

Procedures

The whole procedure included two tasks: resting state, and the finger tapping task (Figure 1A). All participants were first requested to undergo the resting state recording session, during which they were asked to keep their eyes on the fixation cross on the screen, and stay relaxed for 10 minutes. There was 1 minute break between resting state and finger tapping task. During the finger tapping task, participants were presented 11 auditory tones, and they were instructed to tap following the rhythm of the tone. Then, they were instructed to tap according to the rhythm they had learnt without any cues in following 10 minutes (Figure 1A) (1).

According to the previous literature, the spontaneous motor tempo of finger tapping is around 0.6s, meaning the ITI is around 0.6s if no external speed instruction is given to participants (1, 2). Moreover, the performance is best (with the lowest variance and highest synchronization across tapings) around ITI equals to 0.6s (1). Accordingly, fast and slow tapping speed were set to these two tempos. For fast finger tapping, the presumed standard interval (SI) was set as 0.8s to meet the requirement of both closed to the spontaneous tempo as well as including slow EEG cycles like theta. The SI for slow finger tapping was set as 1.9s which is significantly slower than 0.8s (1) and cannot be divided evenly by 0.8s (as to avoid any effects of harmonic frequencies) (Figure 1C). Finally, both resting state and finger tapping tasks (both fast and slow speeds) were further

presented twice, once with thought probes task and once without thought probe tasks as shown in Figure 1A.

Thought probes

Thought probes are widely used in the study of mind wandering as they provide a method to detect subjective states of consciousness (3–7). Thought probes are usually presented randomly during an ongoing task to detect participants' instantaneous thoughts. In this study, 20 thought probes were presented in each task (resting state and finger tapping, Figure 1A), with intervals randomized from 5s to 65s, with jittered 10s intervals. Two questions were asked each time a thought probe was presented to assess the two thought dimensions (e.g., *task relatedness, or, orientation*):

1. Task relatedness: off vs. on task thought:

(In resting state): Are you keeping your eyes on '+'?

(In task): are you focused on the task?

2. Orientation: internal vs. external thought:

Is your thought externally or internally oriented?

We used different questions to assess task-relatedness during both resting state and the finger tapping task because, in the resting state, participants had no tasks to focus on, making it irrelevant to ask if their thoughts were on the 'task'. The scores of thought probes were separated by a visual analog scale (VAS) from 0 to 100. Participants were requested to choose a score according to their state just prior to each thought probe. Higher scores indicated thoughts that are more on-task and more externally-oriented (Figure 1B).

Precision Error

The Precision error (PE) was used in this study to measure the performance of finger tapping (1, 8, 9). PE captures the deviation between the mean of real ITI and the SI by using the ratio between them: $PE = 1 - \frac{ITI}{SI}$; The closer the PE is to zero, the more it indicates a precise replication of the given SI (Figure 1D). Prior to computing the correlation between thought VAS scores and PE, we calculated the absolute value of PE to provide a more stable metric for assessing the relationship with thought dimensions (Figure 2A).

EEG recording and preprocessing

EEG data were collected and preprocessed following the same procedure in both datasets. EEG data was collected from a 64-channel BrainAmp amplifier (Brain Products, Munich, Germany) at a sampling rate of 500 Hz. Electrode Oz was used as the online reference. The impedance for all electrodes was kept below 5 kΩ while the data was recorded. The data preprocessing was conducted using EEGLAB toolbox (10) (http://sccn.ucsd.edu/eeglab/) which is a freely available, open source MATLAB-based package for EEG data analysis. The EEG signals were re-referenced off-line to the average of all electrodes. The signals were band-pass zero-phase FIR filtered at 1-50 Hz. Bad channels were excluded by clean_rawdata plug-in provided in EEGLAB and default settings are used. All stationary artifacts, especially eye movements (blinks and saccades), were reduced by using Independent Component Analysis (ICA) and excluded using the MARA plug-in in EEGlab (11, 12).

EEG analysis

Topographic similarity

Finger tapping task. In order to evaluate temporal integration, we measured topographic similarity, i.e., whether the past tapping shares the similar topography with the present tapping (13, 14). We first set each finger tap as the onset of interest (0ms) and segmented the EEG signals into epochs from -1200ms to 1200ms. For each time point, we averaged the signals within a 50ms window before and after and calculated the corresponding topographic map. Then, for each finger tap, we calculated the topographic similarity between the topographic maps at each time point within the -1200ms to 1200ms window and the topographic map at the corresponding time point of the previous finger tap. Topographic similarity was measured by calculating cosine similarity: $Topographic\ similarity = \frac{V_{n-1} \cdot V_n}{\|V_{n-1}\| \times \|V_n\|}$, in which V_{n-1} represents the vector of the previous tapping topography, and V_n represents the vector of the present tapping topography (Figure 3A).

If the topographic similarity is positive, it indicates that the current topography resembles the previous one, meaning there is a positive interaction between the past and present. If the topographic similarity is close to zero, it suggests that the current topography is unrelated to the previous topographies, indicating a low past-present interaction. If the topographic similarity is negative, it means the current topography has an opposite pattern to the previous one, reflecting a negative interaction between the past and present (Figure 3B).

Baseline in resting state. We also calculated the topographic similarity in the resting state as the baseline for each participant. This was done to obtain each participant's baseline, that is, the expected topographic similarity at standard intervals (fast condition = 0.8s, slow condition = 1.9s) in the absence of a task. The calculation process is similar to that used in the finger-tapping condition, with only one difference: the resting-state signal was epoched at standard intervals, with 0.8s epochs for the fast condition and 1.9s epochs for the slow condition. For each epoch, we

calculated its topographic similarity with the corresponding time points of the previous epoch. Finally, all the epoch values at each timepoint were averaged to obtain the baseline topographic similarity for each participant (supplementary figure 1A & B).

Phase locking value

To investigate the relationship between topographic similarity and phase synchrony, we computed the Phase Locking Value (PLV) between consecutive tapping events, following the method introduced by Lachaux and colleagues (15). PLV quantifies the degree of phase synchronization between two neural signals across repeated measurements—in this case, between adjacent tapping intervals. By assessing PLV in this context, we aimed to determine whether higher topographic similarity is associated with increased inter-tap phase synchrony.

The instantaneous phase of each EEG signal was extracted using the Hilbert transform. For each pair of signals, the phase difference was computed at every time point across trials. These phase differences were converted into complex exponentials, averaged across trials, and then normalized to yield the PLV, as defined by the following formula:

$$PLV(t) = \left| \frac{1}{N} \sum_{n=1}^{N} e^{i(\phi_1(t,n) - \phi_2(t,n))} \right|$$

where $\phi_1(t,n)$ and $\phi_2(t,n)$ denote the instantaneous phase of the two signals at time t for trial n, and *N* is the number of trials. This yielded a time-resolved PLV curve that characterizes phase coherence across trials. To correspond with the analysis of topographic similarity, we averaged the PLV values across all electrode sites after computing them at each site, resulting in a scalp-wide PLV measure.

Phase shuffling

To further test whether topographic similarity is fundamentally phase-based, we performed phase shuffling on the EEG time series (16). This method generates surrogate signals that preserve the spectral power distribution while disrupting the temporal structure (17). Each time series was first transformed into the frequency domain via fast Fourier transform (FFT). The amplitude spectrum was preserved, while the phase spectrum was randomly shuffled under the constraint of Hermitian symmetry, ensuring that the inverse transform yields a real-valued signal. Specifically, random phase values were drawn uniformly from $[-\pi, \pi]$, for the positive frequencies, and the corresponding negative frequency components were assigned their complex conjugates. The inverse FFT was then applied to reconstruct a time-domain surrogate signal that retains the original power spectrum but lacks the original phase relationships. This approach provides a stringent control for testing the temporal specificity of phase-based neural metrics.

After phase shuffling, PLV and topographic similarity were recalculated. The correlations between PLV, topographic similarity, and both PE and thought ratings were then recomputed using the

same procedures as in the non-shuffled condition. To assess the similarity between topographic similarity and thought following phase shuffling, we not only shuffled the EEG signals from both the fast and slow tapping conditions, but also shuffled the EEG signals from the resting state. We then computed the difference between tapping and resting states and examined its correlation with thought. This approach ensured maximal consistency with the original (non-shuffled) analysis pipeline.

Event-related spectral perturbation

As a control and comparison, we also analyzed standard EEG indices commonly used in studies of thought and attention, such as event-related spectral perturbation (ERSP) (18, 19). Following previous literature, we selected three standard electrode sites—Fz, Cz, and Pz—for analysis (20, 21). Baseline correction was performed using the −800 to −100 ms window prior to each tapping event. Consistent with the topographic similarity analysis, each epoch was time-locked to the tapping onset (0 ms) and spanned from −800 ms to 800 ms. To assess condition differences, we applied a cluster-based permutation test to the ERSP data across the −800 to 800 ms time window and the 1–40 Hz frequency range (1000 permutations, cluster-forming threshold $|t| > 2.0$). Additionally, we identified time–frequency clusters whose power values were significantly correlated with precision error (PE) and subjective thought ratings (VAS scores) (1000 permutations, cluster-forming threshold $|r| > 0.2$).

Statistical analysis

Trial-based correlation

We conducted a trial-based analysis rather than a subject-based analysis to examine the relationship between thought, PE, and topographic similarity. Specifically, we aggregated trial data from all participants and assessed the correlation between the trial-based thought VAS scores and the respective trial-based indices of both behavioral (PE) and neural (topographic similarity) measures. Such a single trial-based approach is commonly used in other domains (22, 23) which we extend and apply here to the single thought probes: our focus was mainly on neural (topographic similarity) and behavioral (PE) changes related to states of the thoughts themselves rather than to the subjects' trait features exhibiting those thoughts. This was further reflected in our usage of a continuous rating of thoughts with a continuum between the extremes of internal/external and on/off task: this allowed for a more fine-grained assessment of the single thought probes than the usual binary (on vs off, internal vs external) choice. This is also reflected in the distributions of the thought VAS scores, which did not exhibit a bimodal pattern but rather a continuum within and across participants (Supplementary Figure 2). Unlike the binary distribution often assumed in previous studies using thought probes (5, 7), our findings highlight a continuous nature of thought states thus confirming the usage of a continuous VAS rather than the binary choice. Our primary focus was thus on the overall patterns of variation between the different single trial thought probes across subjects, rather than inter-subject differences (5, 24).

After pooling the single trial thought probes across participants, we averaged the trial values corresponding to each segment of the VAS score (e.g., 0–1, 1–2, 2–3, etc.) for several reasons: 1. to mitigate the risk of overly significant correlations caused by the large number of single trials; 2. to reduce noise stemming from the inherent subjective nature and imprecision of the participants' VAS ratings, as segment averaging allows for smoothing out the variability related to inter-individual differences; 3. to equalize the weighting of each VAS score segment and prevent biases due to uneven distribution of trial counts across segments (22, 23). We further selected values with z-scores between -3 and 3 for Pearson correlation analysis, minimizing the influence of outliers while ensuring the data remained representative and sensitive to overall trends. Finally, we conducted analogous single-based analyses for both behavioral (PE) and neural (topographic similarity) measures to allow for their correlation with the single trial thought probes.

Calculation of the thought's impact on topographic similarity.

The resting state serves as a baseline for subsequent task-related activities with the former modulating the latter (25–27). Consequently, internal cognition associated with the resting state (28, 29) may carry over to the subsequent task states, potential confounding task-related measures. To control for such carry-over effects, we calculated topographic similarity in the resting state with the same time intervals (0.8s or 1.9s) used in the two task states respectively (fast and slow), a method analogous to the 'pseudo-trial' approach described by Huang and colleagues (25); these resting state results were then subtracted from the task states measures, isolating the task-specific effects of topographic similarity. This approach allowed us to differentiate between task-related cognitive processes and those carried over from the resting state, while also accounting for the interaction of timescales (slow, fast) with task-specific thoughts.

This allowed us to calculate the correlation between the task-rest difference of topographic similarity and the thought dimensions (task-relatedness and thought orientation) in a trial-based way (as described above). The absolute value of the correlation coefficient from these correlations served as the measure of thought's impact on topographic similarity (supplementary figure 1B).

To verify that the impact of thought on task-related topographic similarity truly reflects the influence of thought, rather than the differences caused by the given tapping intervals themselves, we conducted partial correlation in which the difference between ITI and SI was included as a covariate to control for the effects of the SI given in the slow and fast tapping tasks. If the results of this partial correlation, after accounting for the covariate, remain consistent with the original findings of the thoughts' impact on topographic similarity, it would confirm that the observed effects are due to the thoughts themselves rather than merely being related to the given tapping intervals during the tapping behavior.

Comparing thought impact on topographic similarity between thought dimensions

We performed 1000 bootstrap resamples on segmented trials. In each resample, we calculated the

thought impact on topographic similarity for both thought dimensions and then subtracted the one for the thought orientation dimension from the one for the task-relatedness dimension. Finally, we used a one-sample t-test to compare the distribution of the 1000 resampled differences to 0. If the result was significantly greater than 0, it indicated that task-relatedness has a stronger impact on topographic similarity, meaning that the task-relatedness dimension contributed more to the topographic similarity. Conversely, if the result was significantly less than 0, it indicated that thought orientation has a stronger impact, meaning that the thought orientation dimension contributed more to the topographic similarity (Figure 4C).

We also performed 1000 times permutation test on the difference of the impacts of different thought dimensions on segmented trials following the procedure proposed by Maris and colleagues (30). To do so, we calculated the difference of thought impact on topographic similarity following the same procedure as above as the 'observed difference', then we applied 1000 times of permutation, and got the distribution of 'permutation difference'**.** if the observed difference is significantly larger than the permutation difference, that is, the difference between the impact of task relatedness and orientation is significantly larger than the randomized level, i.e., 0, we can infer that the **task relatedness's** impact on topographic similarity is significantly higher than **orientation's impact**; on the contrary, if the observed difference is significant lower than the permutation difference, orientation's impact is significantly higher than task relatedness's impact.

Linear Mixed Models and Generalized Additive Models

To further control for inter-individual variability and to deepen our interpretation of the observed double dissociation effects, we conducted follow-up analyses using linear mixed-effects models (LMMs). As a preprocessing step, trials with values exceeding three standard deviations from the mean were excluded to minimize the influence of extreme outliers. The remaining data were then binned within each participant based on their thought dimension scores (task-relatedness and orientation) to reduce intra-subject noise and facilitate smoother trend estimation.

Subsequently, LMMs were constructed to evaluate the relationship between the thought dimensions and either behavioral precision error (PE) or the neural measure of topographic similarity. To assess potential nonlinear associations between thought scores and these outcome variables, we additionally employed Generalized Additive Models (GAMs). Specifically, GAMs were fitted to model the nonlinear trends between PE or topographic similarity and the continuous visual analog scale (VAS) ratings for each thought dimension. To isolate the linear component of these relationships, we extracted the residuals from the GAMs and re-entered them into LMMs.

Moreover, to formally test whether the degree of nonlinearity between thought dimensions and outcome measures on behavioral level (PE) differed across fast and slow tapping conditions, we implemented interaction GAMs. For each predictor (task-relatedness and orientation), data from both tapping conditions were merged and labeled accordingly. We then fitted interaction GAMs using the mgcv package in R (31), incorporating condition-specific smooth terms:

Bayesian Nonlinear Mediation Analysis Using Smooth Functions of Thought Variables

Building on the previously identified nonlinear relationships between thought dimensions and both PE and topographic similarity, we next investigated whether such nonlinearity mediate the effects of thought on behavioral and neural outcomes. Traditional mediation frameworks typically assume linear relationships between the predictor (X), mediator (M), and outcome (Y). However, prior research suggests that neural responses often exhibit nonlinear associations with psychological constructs such as thought (25, 32), which may be poorly captured by linear models. To address this limitation, we employed a Bayesian mediation approach designed to incorporate nonlinear components of thought-related predictors.

Two-Step Modeling Strategy

To capture potential nonlinear mediation effects, we adopted a two-step analytical strategy:

Nonlinear Trend Estimation via GAM: We first used Generalized Additive Models (GAMs) to estimate the smooth, nonlinear relationship between the outcome variable (PE or topographic similarity) and the thought dimension of interest (e.g., task-relatedness or orientation). The smooth term, s(thought) extracted from the GAM reflects the nonlinear transformation of the predictor and serves as a proxy for its latent effect.

Bayesian Mediation Using Smooth-Term as Mediator:

The fitted values of s(thought) were then used as the mediator (M) in a Bayesian mediation model, capturing how the nonlinear component of the thought variable mediates its influence on PE or topographic similarity. In this framework, the raw thought VAS score was treated as the independent variable (X), and PE or topographic similarity served as the dependent variable (Y).

Model Specification and Estimation

We specified a pair of linked regression models within a fully Bayesian framework:

Mediator Model (Path a): $\boldsymbol{M} = \alpha_{\boldsymbol{m}} + \beta_{\boldsymbol{m}} \cdot \boldsymbol{X} + \varepsilon_{\boldsymbol{m}}$

Outcome Model (Paths b and c′): $Y = \alpha_y + \beta_y \cdot X + \beta_M \cdot M + \varepsilon_y$

Indirect Effect: Indirect $= \beta_m \cdot \beta_M$

Total Effect: Total = Indirect $+ \beta_y$

Model parameters were estimated using Markov Chain Monte Carlo (MCMC) sampling, enabling us to obtain posterior distributions for each parameter. This allowed for robust inference based on

mean posterior estimates, 90% Credible Intervals (CIs), and posterior probabilities of direction, thereby quantifying both the magnitude and credibility of indirect and direct pathways. A parameter was considered significant if its 90% CI did not include zero, indicating a consistent direction of effect across the posterior distribution.

Replication dataset results

Behavioral level: Thought orientation occupies longer timescale than task-relatedness.

In the replication dataset, thought orientation scores were significantly lower than task-relatedness across all tasks (resting state: $t(34) = 8.24$, $p < 0.001$; fast FT: $t(34) = 4.59$, $p < 0.001$; slow FT: $t(34) = 4.88$, $p < 0.001$, Supplementary Figure 11). A Friedman test revealed no significant distribution differences across the four thought categories (on/off × internal/external) during rest (Friedman statistic = 0.55, $p = 0.909$, Supplementary Figure 12), though significant differences were present in both tapping conditions (fast: Friedman statistic = 10.00, $p = 0.019$; slow: Friedman statistic = 18.78, $p < 0.001$; Supplementary Figure 12), with no pairwise comparisons reaching significance (all $p > 0.05$; Supplementary Figure 12), Indicating the dissociation between thought dimensions.

PE did not differ between fast and slow tapping ($t(34) = 0.39$, $p = 0.698$; Supplementary Figure 13). The double dissociation between PE and thought was replicated: PE during slow tapping correlated only with orientation ($r(98) = -0.42$, $p_{fdr(3)} < 0.001$), not task-relatedness ($r(95) = 0.01$, $p_{fdr(3)} = 1.000$; Supplementary Figure 14); PE during fast tapping correlated only with task-relatedness ($r(94) = -0.27$, $p_{fdr(3)} = 0.0084$), not orientation ($r(98) = -0.15$, $p_{fdr(3)} = 1.000$; Supplementary Figure 14).

LMMs and GAMs supported these effects. For fast tapping, task-relatedness showed a significant negative effect on PE (Beta = -0.0009, t = -3.89, 95% CI [-0.0013, -0.0004]), which vanished after nonlinear adjustment (adjusted LMM: Beta = -0.0003, t = -1.20, 95% CI [-0.0007, 0.0002]; GAM: edf = 2.29, p = 0.03), replicating the nonlinear masking pattern seen in the main dataset. Orientation showed no initial effect (Beta = -0.0001, t = -0.42, 95% CI [-0.0005, 0.0003]) but revealed a significant negative association after adjustment (adjusted LMM: Beta = -0.0003, t = -1.33, 95% CI [-0.0007, 0.0001]; GAM: edf = 3.75, p = 0.01), consistent with its previously observed linear trend. Under the slow tapping condition, task-relatedness again had a significant initial effect (Beta = -0.0007, t = -3.53, 95% CI = [-0.0011, -0.0003]), which was strongly reduced after nonlinear adjustment (adjusted LMM: Beta = -0.0001, t = -0.50, 95% CI = [-0.0005, 0.0003]; GAM: edf = 6.10, p < 0.0001). Orientation showed a reversed pattern: initially negative (Beta = -0.0007, t = -3.35, 95% CI [-0.0011, -0.0003]), but significantly positive after controlling for nonlinearity (adjusted LMM: Beta = 0.0005, t = 2.64, 95% CI [0.0001, 0.0009]; GAM: edf = 1.00,

p < 0.0001), reaffirming its robust linear role. See Supplementary Table 10 for the results.

Bayesian mediation analyses supported these findings. Task-relatedness showed significant negative indirect effects on PE in both tapping conditions (fast: -0.13, CI [-0.21, -0.04]; slow: -0.11, CI [-0.15, -0.08]), while direct effects were non-significant, confirming a suppression effect. For orientation, a modest positive indirect effect emerged in the fast condition (0.03, CI [0.02, 0.06]). In the slow condition, a large positive direct effect (0.35, CI [-0.30, 1.11]) and a negative but non-significant indirect effect (-0.54, CI [-1.29, 0.11]) suggested potential suppression, though severe multicollinearity (r = -1.00) limits interpretability. See Supplementary Table 11 & 12 for the results

Interaction GAMs further confirmed condition-specific nonlinear patterns. Task-relatedness showed stronger nonlinearity in slow tapping (edf = 6.46, F = 8.23, p < 0.0001) than in fast tapping (edf = 2.21, F = 2.85, p = 0.043), with a significant condition interaction (F = 9.66, p < 0.0001). Orientation was nonlinear in fast tapping (edf = 3.44, F = 2.98, p = 0.019) but linear in slow tapping (edf = 1.00, F = 19.68, p < 0.0001), with a robust interaction effect (F = 160.62, p < 0.0001). This pattern replicates the double dissociation observed in the main dataset. See Supplementary Table 13 for the results

Taken together, these results indicate that the nonlinear relationship between task-relatedness and PE suppresses its overall effect, with this suppression being more pronounced under the slow tapping condition. In contrast, orientation contributes to PE in a consistent and robust linear fashion, and this linear effect may become more detectable when the influence of task-relatedness is masked by nonlinearity. These findings replicate the main dataset.

Neural level: Topographic similarity is impacted by task-relatedness at short intervals and by thought orientation at long intervals.

Topographic similarity was significantly higher during fast vs. slow tapping (t(34) = 13.54, p < 0.001, Supplementary Figure 15A) and correlated with PE in both fast and slow tapping (fast: r(33) = 0.66, $p_{fdr(1)}$ < 0.001; slow: r(32) = 0.76, $p_{fdr(1)}$ < 0.001, Supplementary Figure 15B). Task-rest difference in topographic similarity also correlated with PE (fast: r(33) = 0.66, p_{fdr} < 0.001; slow: r(32) = 0.74, p_{fdr} < 0.001, Supplementary Figure 15C).

We replicated the double dissociation: in fast tapping, topographic similarity was associated with task-relatedness (r(94) = 0.25, $p_{fdr(3)}$ = 0.026), not orientation (r(98) = 0.11, $p_{fdr(3)}$ = 1.000, Supplementary Figure 15D); in slow tapping, the pattern reversed (orientation: r(91) = -0.46, $p_{fdr(3)}$ < 0.001; task-relatedness: r(95) = 0.13, $p_{fdr(3)}$ = 1.000; Supplementary Figure 15D). Task-relatedness had greater influence than orientation during fast tapping while the reverse was true

for slow tapping (fast: mean =0.11, SD = 0.13, SEM = 0.004, t(98) = 26.61, p < 0.001; slow: mean = -0.12, SD = 0.16, SEM = 0.005, t(98) = 23.35, p < 0.001, Supplementary Figure 15E).

Neural level: thought dimensions impact topographic similarity through nonlinear interaction and phase-based mechanisms

In fast tapping, task-relatedness showed a significant positive effect on topographic similarity (Beta = 0.0006, t = 2.40, 95% CI [0.0001, 0.0011]) that vanished after nonlinear control (Beta = -0.0002, t = -0.83, 95% CI [-0.0007, 0.0003]), with strong nonlinearity confirmed (edf = 4.31, p < 0.0001). Orientation shifted from a weak positive effect (Beta = 0.0002, t = 0.71, 95% CI [-0.0003 0.0007]) to a significant negative association (Beta = -0.0006, t = -2.23, 95% CI [-0.0011, -0.0001]), with moderate nonlinearity (edf = 3.96, p = 0.0001). In slow tapping, task-relatedness was initially non-significant (Beta = 0.0000, t = -0.12, 95% CI [-0.0006 0.0006]) but became more negative after adjustment (Beta = -0.0005, t = -1.55, 95% CI [-0.0011, 0.0001]), with mild nonlinearity (edf = 2.80, p = 0.166). Orientation shifted from weakly positive (Beta = 0.0004, t = 1.18, 95% CI [-0.0002. 0.0010]) to significantly negative (Beta = -0.0007, t = -2.19, 95% CI [-0.0013, -0.0001]), with strong nonlinearity (edf = 4.16, p < 0.0001). See Supplementary Table 14 for the detailed results.

Bayesian mediation analyses confirmed these nonlinear effects. In the fast condition, task-relatedness showed a significant indirect effect (mean = 0.19, [0.12, 0.25]), with non-significant direct (-0.03, [-0.12, 0.06]) and significant total effects (0.16, [0.09, 0.23]). Orientation also showed a significant indirect effect (0.18, [0.12, 0.25]), with non-significant direct (-0.03, [-0.13, 0.07]) and significant total effects (0.15, [0.08, 0.22]). In the slow condition, task-relatedness showed a significant indirect effect (0.14, [0.05, 0.22]), non-significant direct (-0.07, [-0.18, 0.04]), and marginal total effect (0.07, [-0.01, 0.14]). Orientation exhibited significant indirect (0.21, [0.14, 0.28]) and total (0.18, [0.11, 0.26]) effects, with non-significant direct effect (-0.03, [-0.13, 0.07]). See Supplementary Table 15 for the detailed results.

Since the Bayesian mediation analyses did not reveal a clear double dissociation effect, we further employed a permutation test to compare the strength of the indirect effects. The results confirmed a double dissociation in indirect effect strength: task-relatedness exceeded orientation in the fast condition (Δ = 0.0283, p < 0.001), while orientation dominated in the slow condition (Δ = -0.0726, p < 0.001).

ERSP results confirmed beta-band power increases under fast tapping, with multiple significant clusters at Fz (e.g., 12.1–33.2 Hz, -800 to -480 ms, p = 0.001), Cz (e.g., 10.6–35.5 Hz, -944 to -508 ms, p = 0.003), and Pz (e.g., 9.8–33.2 Hz, -944 to -508 ms, p = 0.007). Cluster-based correlations revealed that only fast tapping ERSP clusters correlated with PE (e.g., Fz: 3–40 Hz, -554 to 336 ms, p < 0.001); no significant PE or thought correlations emerged in slow tapping (all p > 0.05). See Supplementary Figure 16-18 for ERSP results.

PLV correlated strongly with topographic similarity (fast: $r(33) = 0.72$, $p < 0.0001$; slow: $r(33) = 0.77$, $p < 0.0001$; supplementary figure 19A). Phase-shuffling abolished PE–similarity correlations (fast: $r(32) = -0.07$, $p = 0.680$; slow: $r(32) = -0.04$, $p = 0.812$; supplementary figure 19B) and most thought-related associations. A single correlation remained between phase-shuffled similarity and task-relatedness under fast tapping ($r(94) = 0.28$, $p_{fdr(3)} = 0.026$; supplementary figure 19C), but this was not observed in the main dataset, confirming that phase structure is essential for thought-related neural dynamics.

Supplementary figures

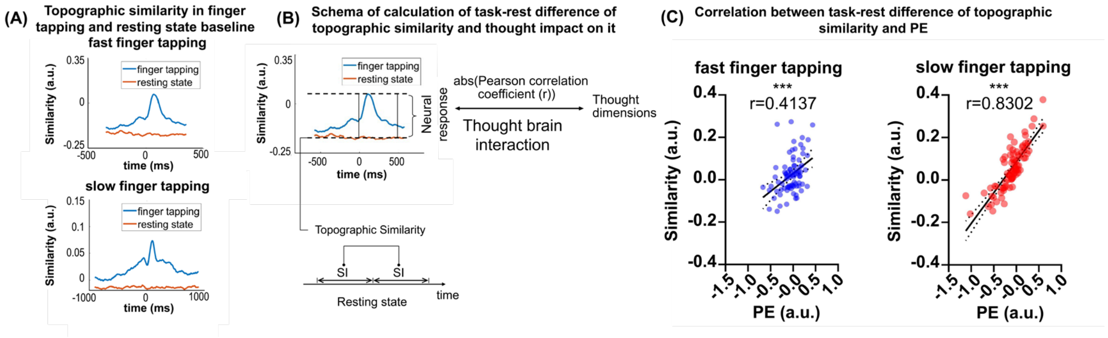

Supplementary figure 1. The calculation of task-rest difference of topographic similarity and its relationship with PE. A) Topographic similarity during finger tapping and resting state baseline. Here we show that the resting state baseline is stable across time, whereas topographic similarity peaks during the post-stimulus period in both fast and slow finger tapping. **B) Schema of calculation of task-rest difference of topographic similarity.** The topographic similarity in the resting state was calculated as the baseline for each participant. The calculation process is similar to that used for the finger tapping condition, with only one difference: the resting-state signal was epoched at SIs, with 0.8s epochs for the fast condition and 1.9s epochs for the slow condition. For each epoch, we calculated its topographic similarity with the corresponding time points of the previous epoch. Finally, all the epoch values at each timepoint were averaged to obtain the resting state's topographic similarity for each participant, e.g. their baseline. Then we calculated the correlation between the task-rest difference of topographic similarity and thought dimensions (task-relatedness and thought orientation) respectively in which we still take the same single trial results (as binned; see methods) of the thought probes as in their correlation with the precision index (see above). We used the absolute value of the correlation coefficient of the correlations as the measure of the thoughts' impact on topographic similarity **C) Correlation between task-rest difference of topographic similarity and PE.** Calculating task-rest difference of topographic similarity, we explored whether topographic similarity remained associated with PE. The results showed that even after subtracting the resting state baseline, the neural responses were still significantly correlated with PE in both fast and slow finger tapping tasks.

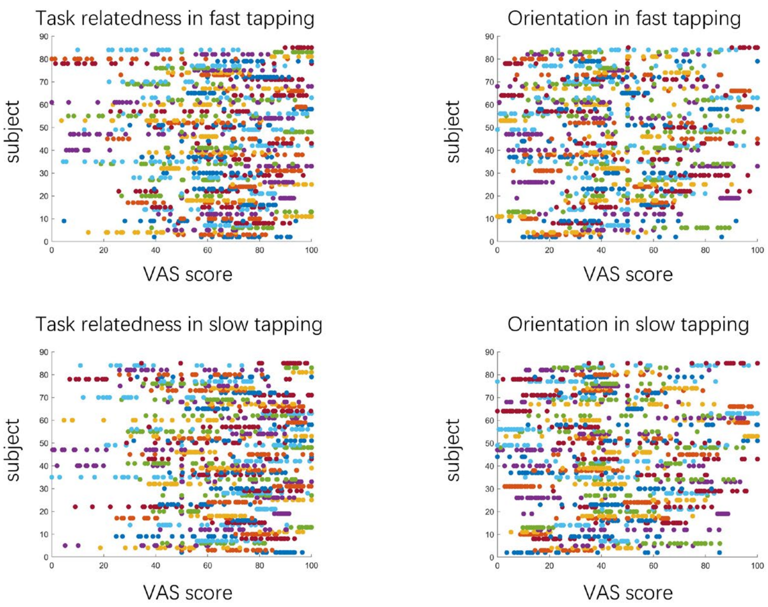

Supplementary figure 2. The VAS scores of all participants. The distributions of the thought VAS scores did not exhibit a bimodal pattern but rather a continuum within and across participants.

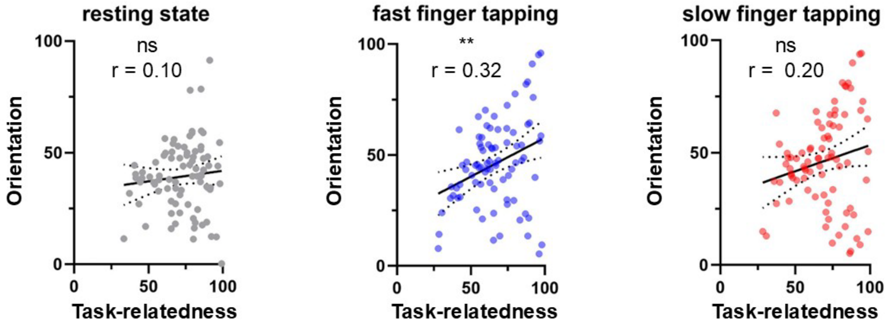

Supplementary Figure 3. Correlation between task-relatedness and thought orientation scores across conditions. Scatter plots showing the relationship between task-relatedness and thought orientation scores in the three experimental conditions: (A) resting state, (B) fast finger tapping, and (C) slow finger tapping. The plots highlight a noticeable association between the two dimensions during the fast finger tapping condition, while no apparent relationship is observed in the resting state or slow finger tapping condition. These results indicate condition-specific differences in the interplay between task-relatedness and thought orientation. Note: ns: none significance; **: p<0.01.

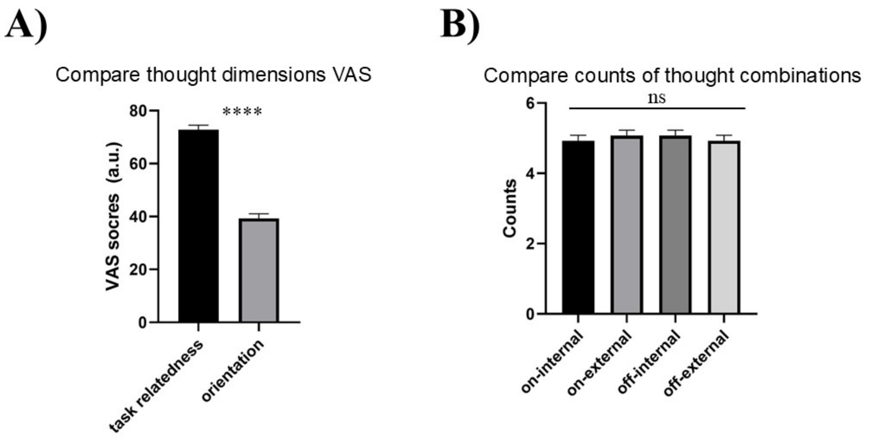

Supplementary Figure 4. Comparison of thought dimension ratings across conditions. (A) Bar plots depicting participants' VAS ratings for task-relatedness (off-task vs. on-task) and thought orientation (internal vs. external) in resting state. The plots reveal that task-relatedness ratings are consistently higher than thought orientation ratings in resting state. **(B) Bar plots illustrate the mean trial counts for the four thought categories (on-task/internal, off-task/internal, on-task/external, and off-task/external) in resting state.** The distribution shows no significant differences during the resting state. Note: ****: p<0.0001; ns: none significance.

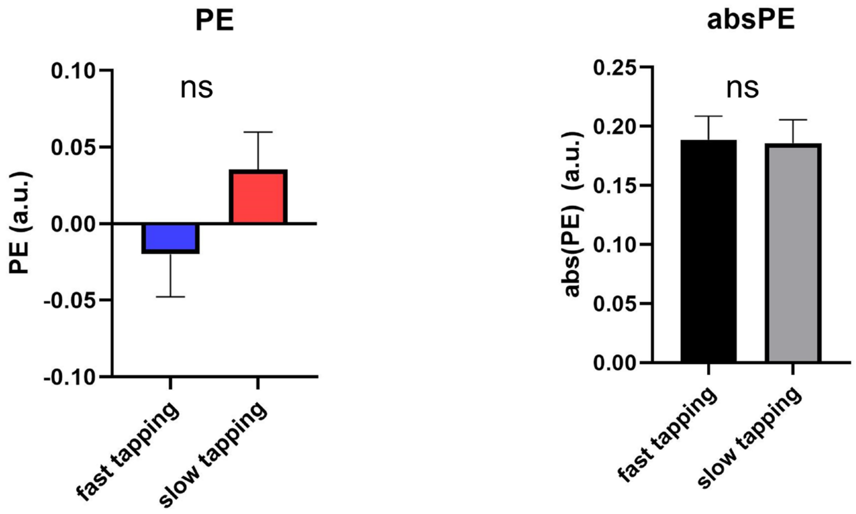

Supplementary Figure 5. Comparison of PE across fast and slow finger tapping conditions. Left figure: Comparison of PE between fast and slow finger tapping tasks. No significant difference was found of PE between fast and slow finger tapping. **Right figure: comparison of the absolute value of PE between fast and slow finger tapping conditions.** The result also showing no significant difference. These findings suggest that the duration of tapping intervals (shorter in fast tapping and longer in slow tapping) does not systematically affect PE. Note: ns: none significance.

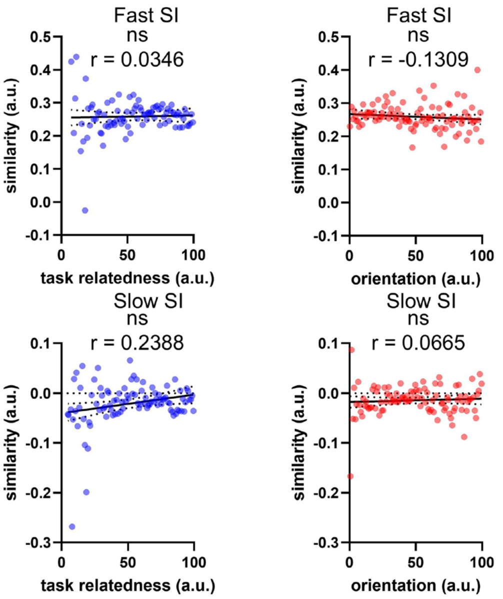

Supplementary Figure 6. Correlation between resting-state topographic similarity and thought dimensions across conditions. Scatter plots illustrating the relationship between resting-state topographic similarity and the two thought dimensions—task-relatedness and thought orientation. No significant correlations were observed for either thought dimension in both conditions (fast SI = 0.8s or slow SI = 1.9s), suggesting that resting-state topographic similarity is not associated with task-relatedness or thought orientation in resting state. Note: ns: none significance.

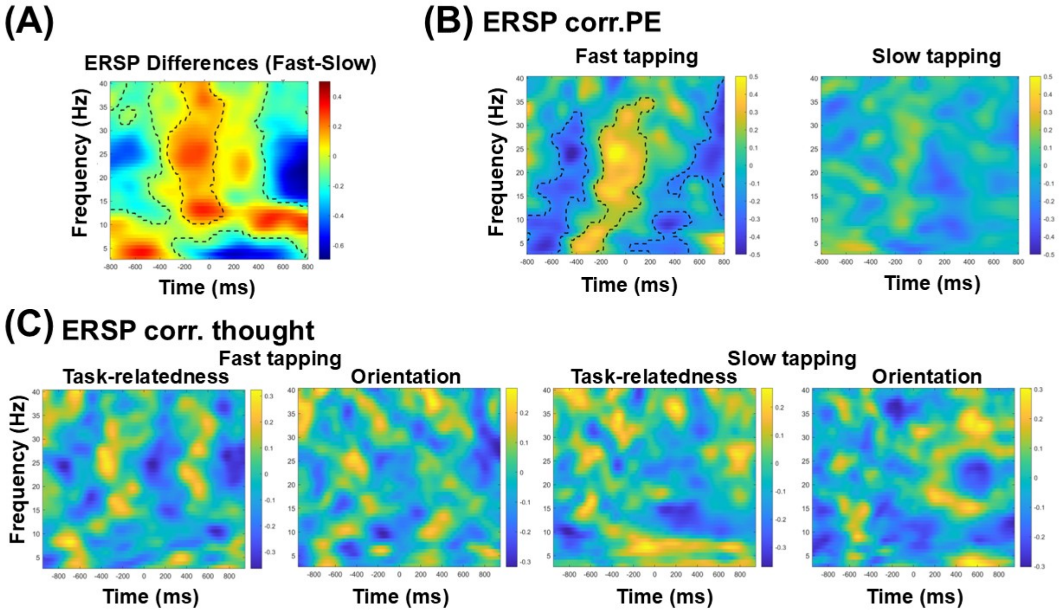

Supplementary figure 7. Time–frequency analysis of ERSP differences and correlations with behavior and thought at Fz. (A) Condition differences in ERSP between fast and slow tapping. Warm colors indicate greater power during fast tapping; cool colors indicate greater power during slow tapping. (B) Correlations between ERSP power and PE under fast (left) and slow (right)

conditions. (C) Correlations between ERSP power and thought probe ratings (task-relatedness and orientation) under fast (left two panels) and slow (right two panels) tapping. In all panels, black dashed contours mark statistically significant clusters identified through cluster-based permutation testing ($p < 0.05$, corrected). Color scales in (A) reflect power differences (fast – slow), while in (B) and (C) they represent Pearson's correlation coefficients (r values).

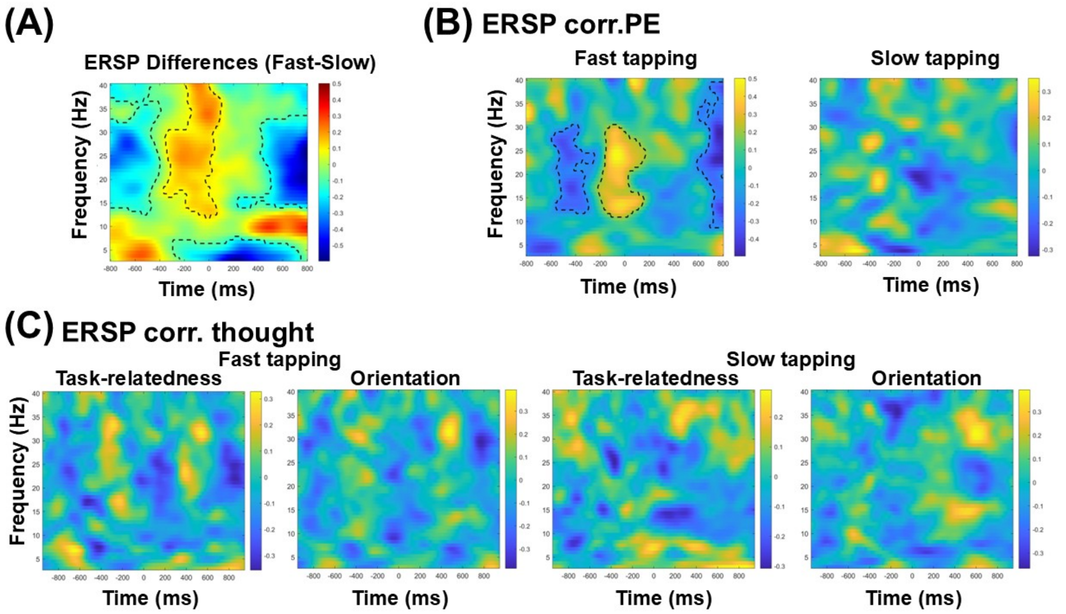

Supplementary figure 8. Time–frequency analysis of ERSP differences and correlations with behavior and thought at Cz. (A) Condition differences in ERSP between fast and slow tapping. Warm colors indicate greater power during fast tapping; cool colors indicate greater power during slow tapping. (B) Correlations between ERSP power and PE under fast (left) and slow (right) conditions. (C) Correlations between ERSP power and thought probe ratings (task-relatedness and orientation) under fast (left two panels) and slow (right two panels) tapping. In all panels, black dashed contours mark statistically significant clusters identified through cluster-based permutation testing ($p < 0.05$, corrected). Color scales in (A) reflect power differences (fast – slow), while in (B) and (C) they represent Pearson's correlation coefficients (r values).

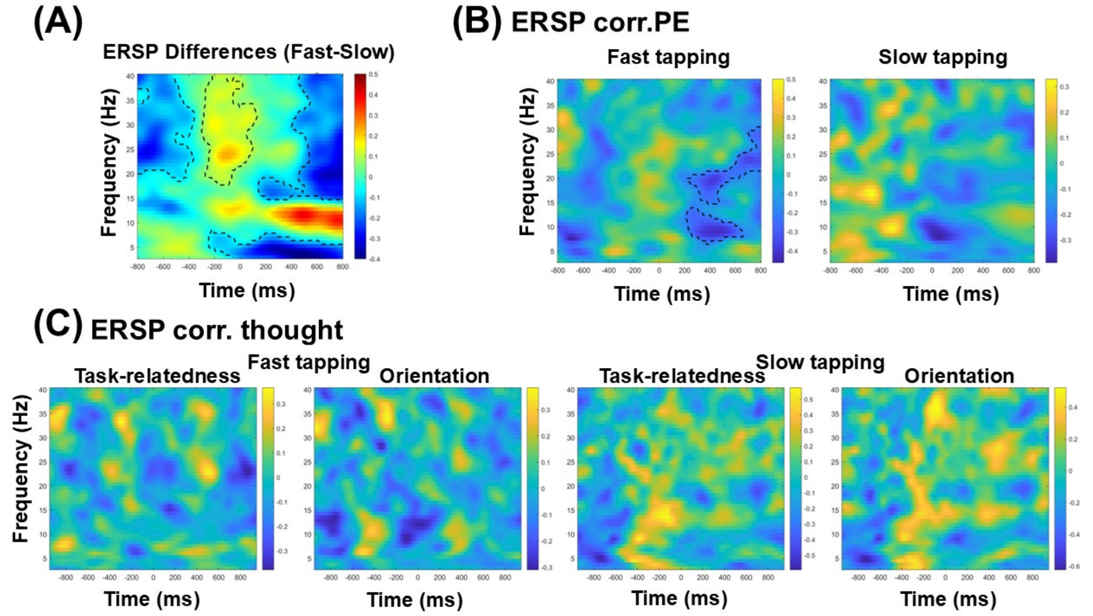

Supplementary figure 9. Time–frequency analysis of ERSP differences and correlations with behavior and thought at Pz. (A) Condition differences in ERSP between fast and slow tapping. Warm colors indicate greater power during fast tapping; cool colors indicate greater power during slow tapping. (B) Correlations between ERSP power and PE under fast (left) and slow (right) conditions. (C) Correlations between ERSP power and thought probe ratings (task-relatedness and orientation) under fast (left two panels) and slow (right two panels) tapping. In all panels, black dashed contours mark statistically significant clusters identified through cluster-based permutation testing ($p < 0.05$, corrected). Color scales in (A) reflect power differences (fast – slow), while in (B) and (C) they represent Pearson's correlation coefficients (r values).

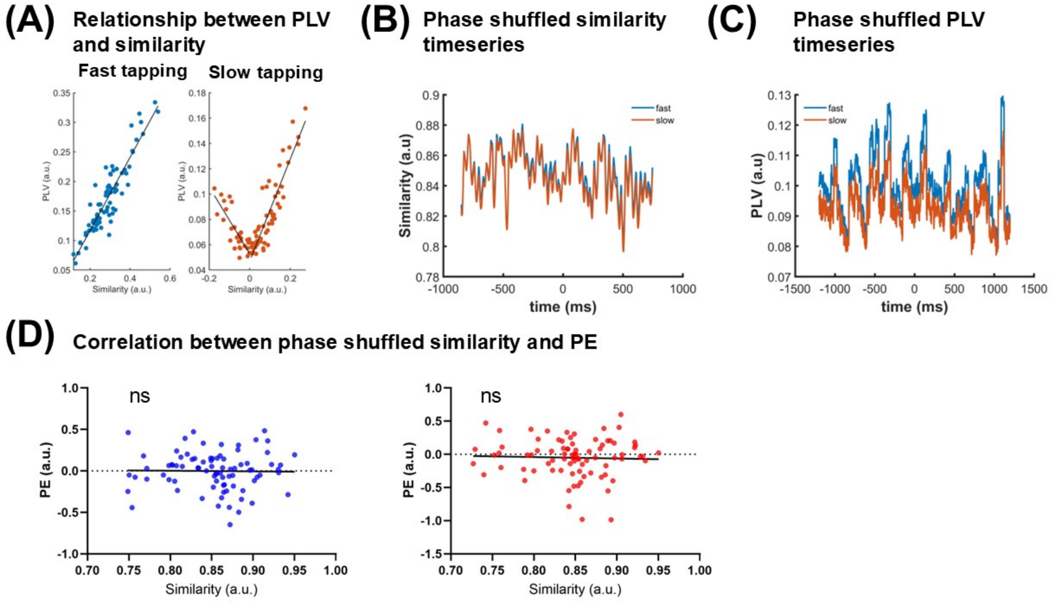

Supplementary Figure 10. Phase based analysis of PLV and topographic similarity. (A) Raw

scatterplot showing the piecewise association between PLV and untransformed topographic similarity under the slow tapping condition, due to the presence of negative similarity values. (B) Topographic similarity time course before and after phase-shuffling, showing elimination of the peak observed in the original data. (C) PLV time course before and after phase-shuffling. The characteristic 0–300 ms post-stimulus peak disappears after shuffling. (D) Correlations between topographic similarity and PE under both tapping conditions vanish after phase-shuffling. Note: *: p<0.05; **: p<0.01; ns: not significant.

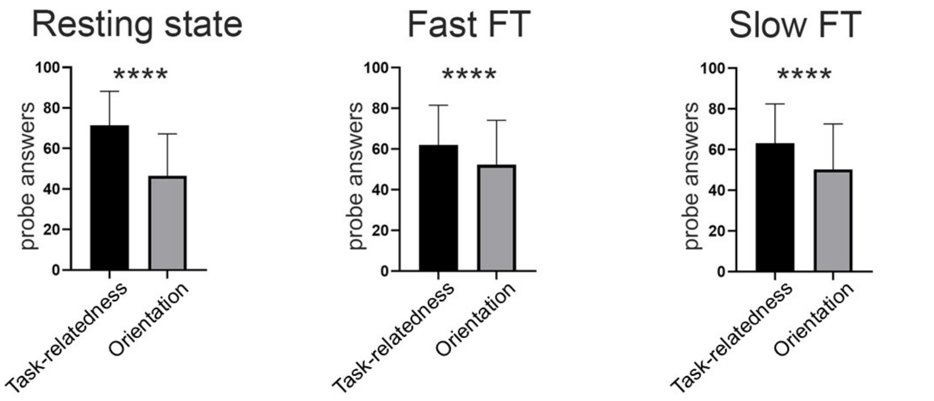

Supplementary Figure 11. Comparison of task-relatedness and thought orientation VAS scores in the replication dataset across conditions. Consistent with the findings from the first dataset, thought orientation scores were significantly lower than task-relatedness scores in all three conditions, demonstrating the replicability of the observed differences between the two thought dimensions. Note: ****: p<0.0001.

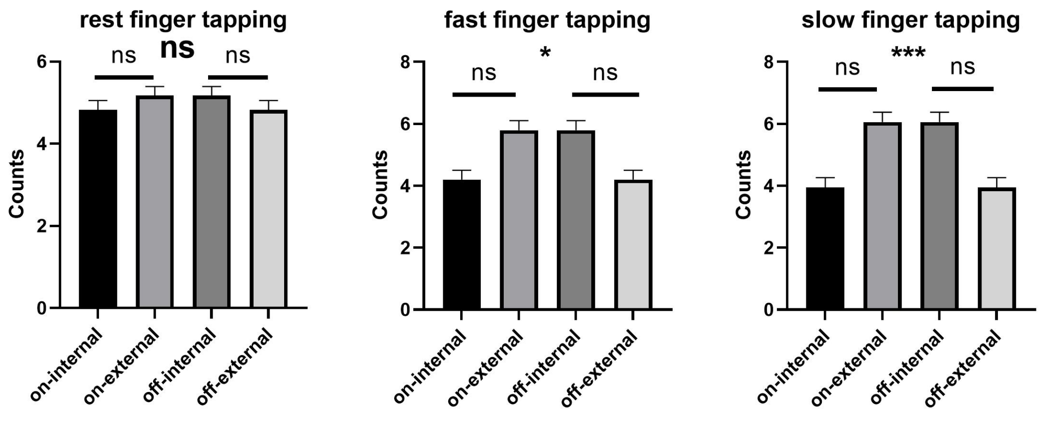

Supplementary Figure 12. Distribution of thought categories across conditions in the replication dataset. Statistical analysis results indicate no significant differences among the four thought categories during the resting state, as revealed by the Friedman test. However, significant

differences were observed among the categories during fast and slow finger tapping conditions. Despite these overall differences, multiple comparison tests showed no significant pairwise differences between any of the four categories in either fast or slow finger tapping. These findings, consistent with the first dataset, confirm that task-relatedness and thought orientation represent distinct cognitive dimensions without strict correspondence between specific thought combinations. Note: ns: none significance. ****: $p<0.0001$.

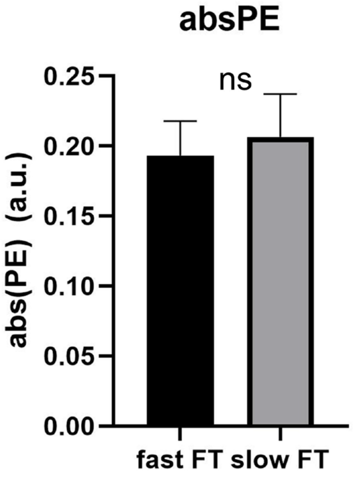

Supplementary figure 13. Comparison of the absolute value of PE between fast and slow tapping in the replication dataset. The absolute value PE was not significantly different between fast and slow FT. Note: ns: none significance.

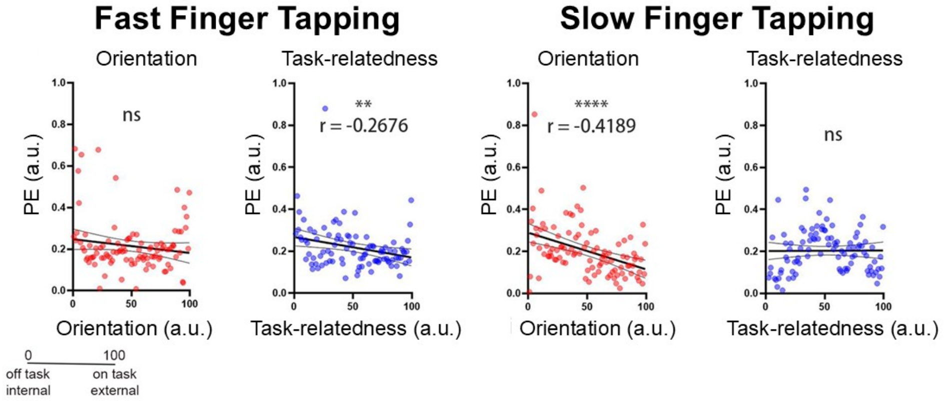

Supplementary Figure 14. Double dissociation of the correlation between PE and thought dimensions across tapping conditions in the replication dataset. In fast FT, PE is significantly correlated with task-relatedness but not with thought orientation, highlighting a selective association with task-relatedness at faster tapping speeds. In slow FT, PE is significantly

correlated with thought orientation but not with task-relatedness, indicating a selective association with thought orientation at slower tapping speeds. These findings replicate the double dissociation observed in the first dataset, further supporting the distinction between task-relatedness and thought orientation as independent thought dimensions. Note: ns: none significance; **: p<0.01; ****: p<0.0001.

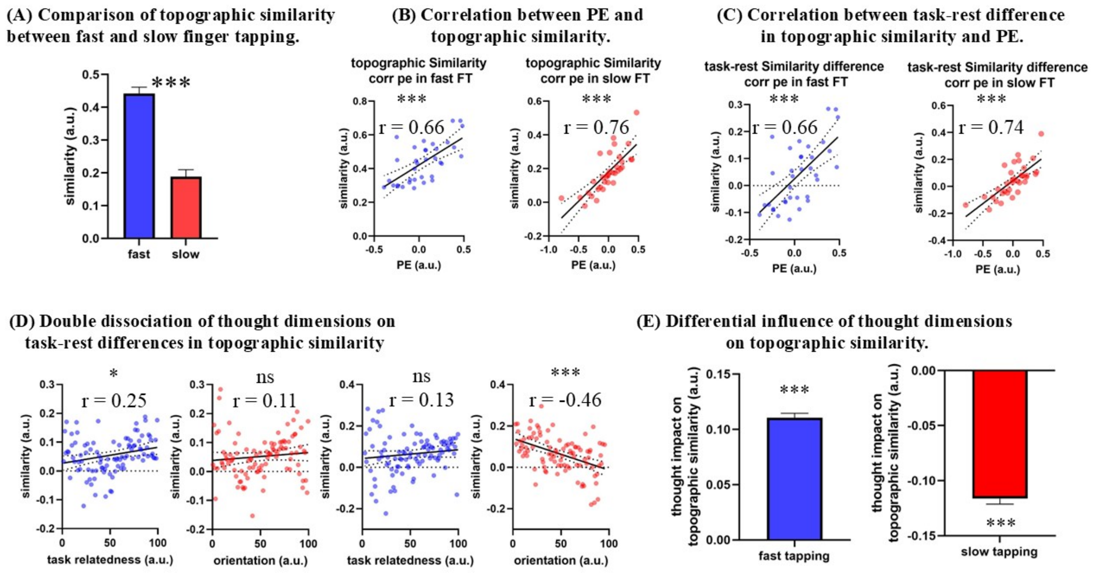

Supplementary Figure 15. Replication of the neural correlates of task-relatedness and thought orientation in the replication dataset. (A) Comparison of topographic similarity between fast and slow finger tapping. Topographic similarity is significantly higher during fast finger tapping (FT) compared to slow FT, replicating previous findings. **(B) Correlation between PE and topographic similarity.** Significant correlations are observed between PE and topographic similarity in both fast and slow FT conditions. **(C) Correlation between task-rest difference in topographic similarity and PE.** The task-rest difference in topographic similarity is significantly correlated with PE in both fast and slow FT conditions, highlighting the impact of PE on neural dynamics during task performance. **(D) Double dissociation of thought dimensions on task-rest differences in topographic similarity.** In fast FT, task-rest differences in topographic similarity are correlated with task-relatedness but not thought orientation. In slow FT, the opposite pattern is observed, with significant correlations only with thought orientation. **(E) Differential influence of thought dimensions on topographic similarity.** During fast FT, task-relatedness exerts a greater influence on topographic similarity, while during slow FT, thought orientation has a stronger effect. These findings replicate and extend the neural-level double dissociation between task-relatedness and thought orientation, further supporting their distinct contributions under different temporal contexts. Note: ns: none significance; ***: p<0.001.

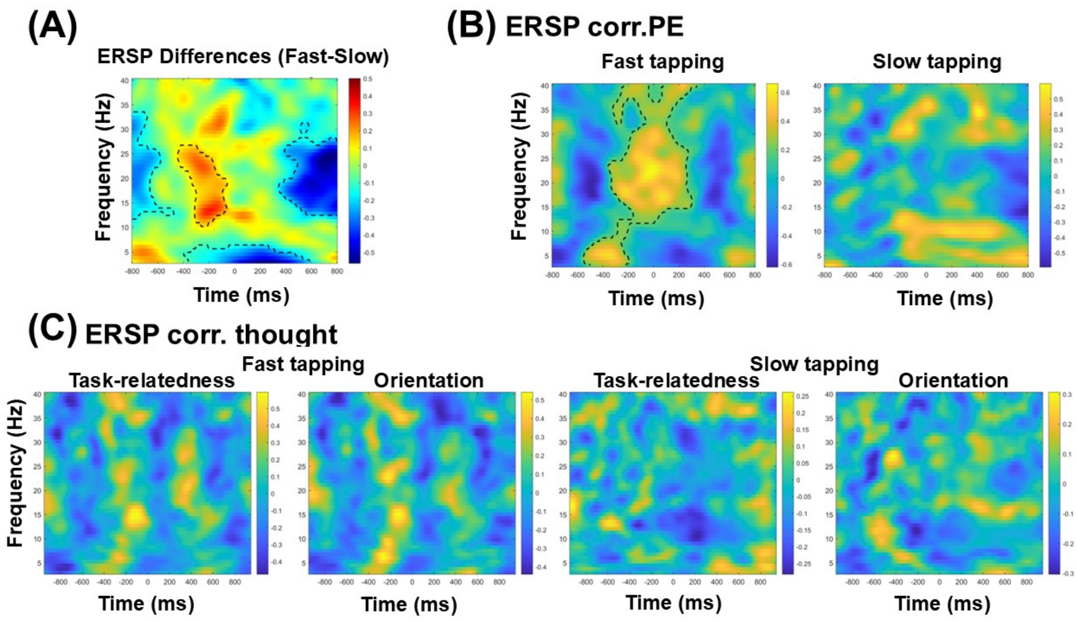

Supplementary figure 16. Time–frequency analysis of ERSP differences and correlations with behavior and thought at Fz on replication dataset. (A) Condition differences in ERSP between fast and slow tapping. Warm colors indicate greater power during fast tapping; cool colors indicate greater power during slow tapping. (B) Correlations between ERSP power and PE under fast (left) and slow (right) conditions. (C) Correlations between ERSP power and thought probe ratings (task-relatedness and orientation) under fast (left two panels) and slow (right two panels) tapping. In all panels, black dashed contours mark statistically significant clusters identified through cluster-based permutation testing ($p < 0.05$, corrected). Color scales in (A) reflect power differences (fast – slow), while in (B) and (C) they represent Pearson's correlation coefficients (r values).

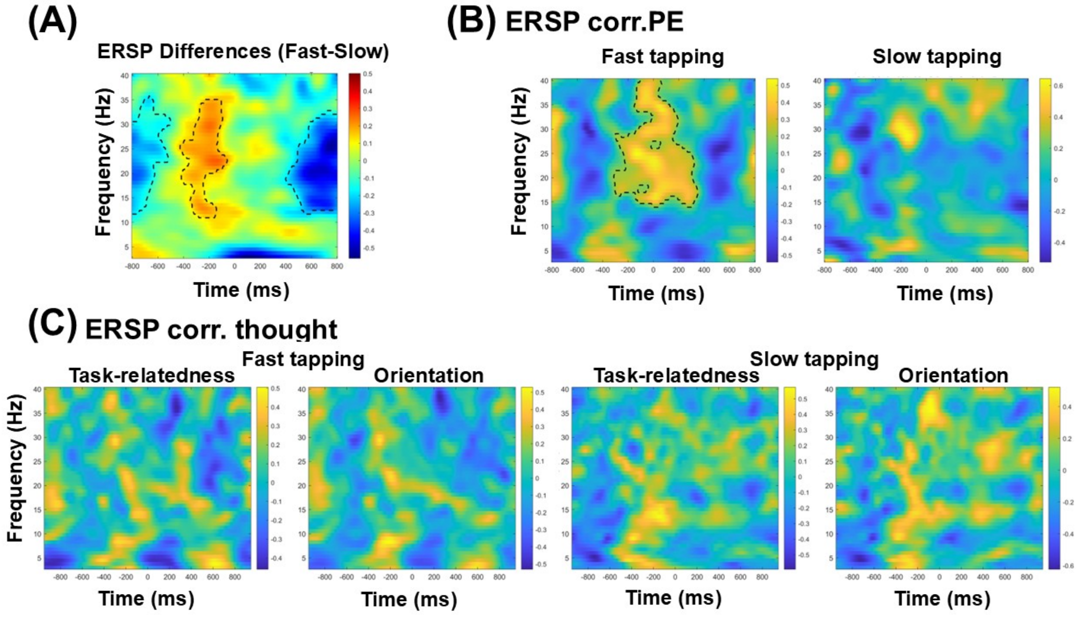

Supplementary figure 17. Time–frequency analysis of ERSP differences and correlations

with behavior and thought at Cz on replication dataset. (A) Condition differences in ERSP between fast and slow tapping. Warm colors indicate greater power during fast tapping; cool colors indicate greater power during slow tapping. (B) Correlations between ERSP power and PE under fast (left) and slow (right) conditions. (C) Correlations between ERSP power and thought probe ratings (task-relatedness and orientation) under fast (left two panels) and slow (right two panels) tapping. In all panels, black dashed contours mark statistically significant clusters identified through cluster-based permutation testing ($p < 0.05$, corrected). Color scales in (A) reflect power differences (fast – slow), while in (B) and (C) they represent Pearson's correlation coefficients (r values).

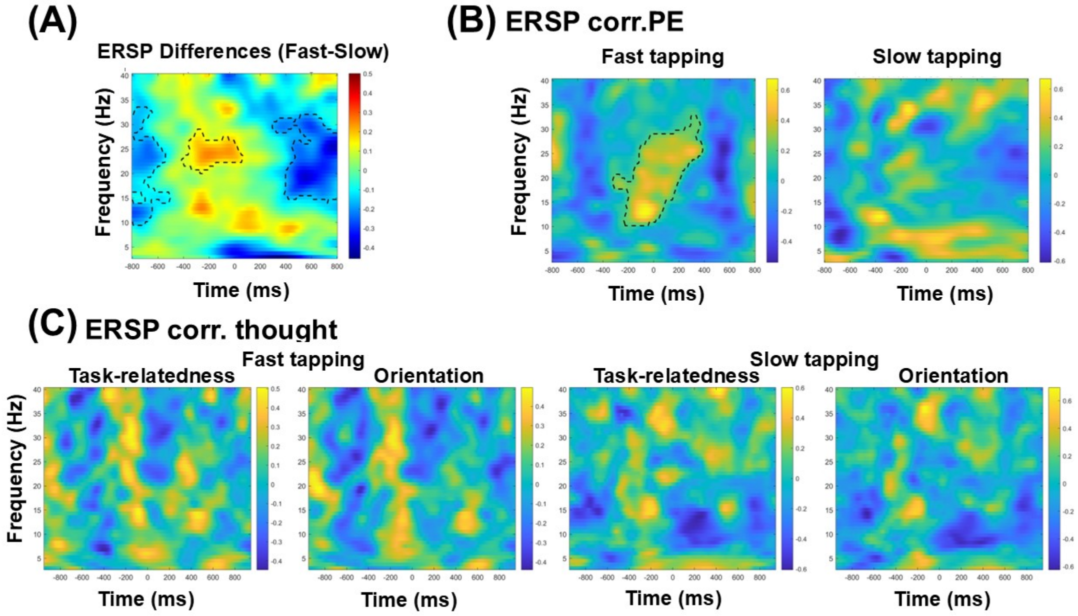

Supplementary figure 18. Time–frequency analysis of ERSP differences and correlations with behavior and thought at Pz on replication dataset. (A) Condition differences in ERSP between fast and slow tapping. Warm colors indicate greater power during fast tapping; cool colors indicate greater power during slow tapping. (B) Correlations between ERSP power and PE under fast (left) and slow (right) conditions. (C) Correlations between ERSP power and thought probe ratings (task-relatedness and orientation) under fast (left two panels) and slow (right two panels) tapping. In all panels, black dashed contours mark statistically significant clusters identified through cluster-based permutation testing ($p < 0.05$, corrected). Color scales in (A) reflect power differences (fast – slow), while in (B) and (C) they represent Pearson's correlation coefficients (r values).

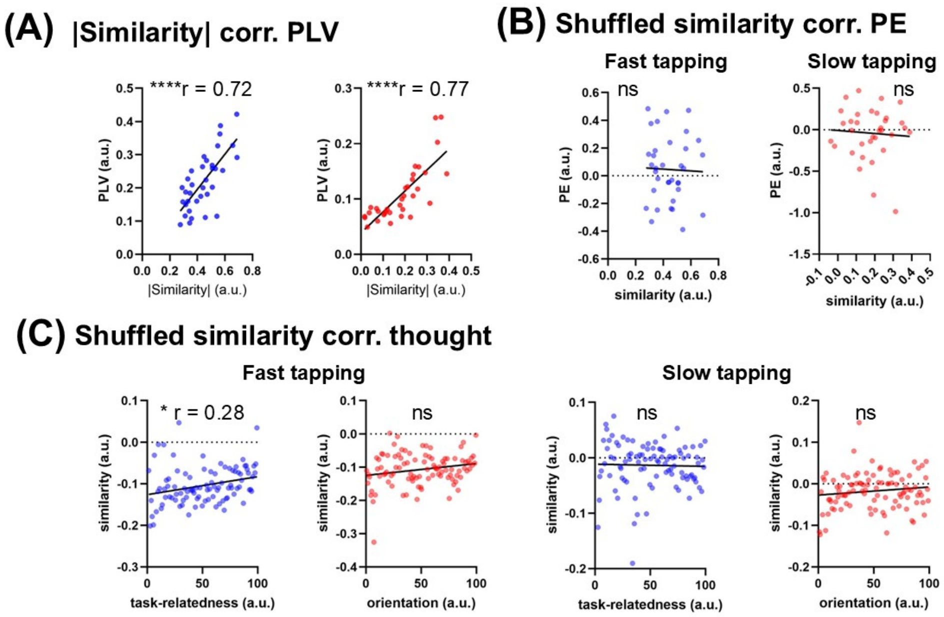

Supplementary Figure 19. Phase-based validation of topographic similarity using correlation and phase-shuffling analyses. (A) Scatterplots showing strong positive correlations between PLV and the absolute value of topographic similarity under both tapping conditions. (B) After phase-shuffling, the correlation between topographic similarity and PE is abolished under both conditions. (C) Correlation plots between phase-shuffled topographic similarity and thought dimensions. A significant correlation with task-relatedness was observed only under fast tapping, while all other associations were non-significant. Note: *: $p < 0.05$;

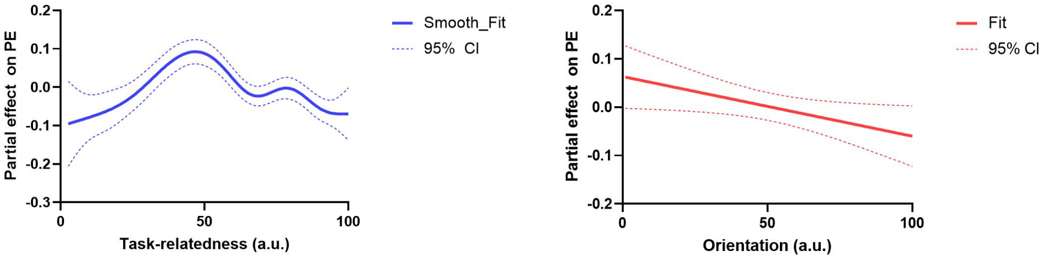

Supplementary Figure 20. An example of GAM fits illustrating the nonlinear relationship between task-relatedness and PE and linear relationship between orientation and PE.

Supplementary tables

Supplementary Table 1. Fast tapping Friedman test multiple comparison results

Comparison	Rank sum diff.	Adjusted P Value
on-internal vs. on-external	-34	0.253
on-internal vs. off-internal	-34	0.253
on-internal vs. off-external	0	>0.9999
on-external vs. off-internal	0	>0.9999
on-external vs. off-external	34	0.253
off-internal vs. off-external	34	0.253

Supplementary Table 2. Slow tapping Friedman test multiple comparison results

Comparison	Rank sum diff.	Adjusted P Value
on-internal vs. on-external	-38	0.1389
on-internal vs. off-internal	-38	0.1389
on-internal vs. off-external	0	>0.9999
on-external vs. off-internal	0	>0.9999
on-external vs. off-external	38	0.1389
off-internal vs. off-external	38	0.1389

Supplementary table 3. Details of LMMs and GAMs

Fast Tapping

LMM raw values

Predictor	Beta	SE	t	CI_lower	CI_upper
Task relatedness	-0.0006	0.0003	-1.8659	-0.0011	0.0000
Orientation	0.0000	0.0003	0.0432	-0.0005	0.0005

GAM

Predictor	edf	F_value	p_value
Task relatedness	2.2302	2.2635	0.1013
Orientation	1.0003	13.8395	0.0002

LMM nonlinearity controlled

Predictor	Beta	SE	t	CI_lower	CI_upper
Task relatedness	0.0001	0.0003	0.2532	-0.0005	0.0007
Orientation	0.0010	0.0003	3.8337	0.0005	0.0015

Slow Tapping

LMM raw values

Predictor	Beta	SE	t	CI_lower	CI_upper
Task relatedness	-0.0010	0.0003	-2.8537	-0.0017	-0.0003
Orientation	-0.0002	0.0003	-0.8047	-0.0009	0.0004

GAM

Predictor	edf	F_value	p_value
Task relatedness	5.0273	3.8886	0.0009
Orientation	1.0369	16.9425	0.0000

LMM nonlinearity controlled

Predictor	Beta	SE	t	CI_lower	CI_upper
Task relatedness	0.0000	0.0003	-0.0212	-0.0007	0.0007
Orientation	0.0010	0.0003	3.3622	0.0004	0.0016

Supplementary table 4. Bayesian Mediation Analysis of Task-Relatedness and Orientation Effects on PE

Condition	Thought dimension	Variable	Mean	q5	q95	$\hat{r}$
Fast	Task-relatedness	Indirect	-0.11	*-0.20*	*-0.04*	1.00
		Direct	0.04	-0.07	0.15	1.00
		Total	-0.07	*-0.15*	*0.00*	1.00
	Orientation	Indirect	-0.59	-1.84	0.56	2.82
		Direct	0.41	-0.74	1.67	2.81
		Total	-0.18	*-0.25*	*-0.10*	1.03
Slow	Task-relatedness	Indirect	-0.12	*-0.16*	*-0.08*	1.00
		Direct	0.02	-0.06	0.11	1.00
		Total	-0.1	*-0.17*	*-0.02*	1.00
	Orientation	Indirect	-0.35	-0.81	0.13	1.02
		Direct	0.35	-0.14	0.80	1.02
		Total	-0.009	*-0.012*	*-0.005*	1.00

Note: Effects whose 90% credible intervals do not include zero are interpreted as statistically meaningful (*italicized* in parentheses).

Supplementary table 5. r values of Pearson Correlations Among Orientation, Smooth Term s(orientation), and PE

	orientation	s(orientation)	PE
orientation	1.00	-1.00	-0.17
s(orientation)	-1.00	1.00	0.17
PE	-0.17	0.17	1.00

Supplementary table 6. Interaction GAM Analysis Comparing Nonlinear Contributions of Thought Dimensions on PE Across Conditions

Predictor	F	p
Task-Relatedness (T)	2.45	0.027
Orientation (O)	0.64	0.493

Supplementary table 7. Nonlinear modeling of the relationship between thought dimensions and topographic similarity across tapping conditions.

**Fast Tapping**					
**LMM raw values**					
Predictor	Beta	SE	t	CI_lower	CI_upper
Task relatedness	0.0004	0.0002	2.5689	0.0001	0.0008
Orientation	0.0003	0.0001	1.9406	0.0000	0.0005
**GAM**					
Predictor	edf	F_value	p_value		
Task relatedness	3.7448	4.2763	0.0011		
Orientation	1.0020	4.2675	0.0390		
**LMM nonlinearity controlled**					
Predictor	Beta	SE	t	CI_lower	CI_upper
Task relatedness	0.0000	0.0002	-0.2391	-0.0004	0.0003
Orientation	0.0000	0.0001	-0.0301	-0.0003	0.0003
**Slow Tapping**					
**LMM raw values**					
Predictor	Beta	SE	t	CI_lower	CI_upper
Task relatedness	0.0001	0.0002	0.7033	-0.0002	0.0005
Orientation	-0.0002	0.0002	-1.0705	-0.0005	0.0001
**GAM**					
Predictor	edf	F_value	p_value		
Task relatedness	1.0004	1.9648	0.1612		
Orientation	3.1511	1.0924	0.3683		
**LMM nonlinearity controlled**					
Predictor	Beta	SE	t	CI_lower	CI_upper
Task relatedness	-0.0001	0.0002	-0.5018	-0.0004	0.0003
Orientation	-0.0002	0.0002	-1.1412	-0.0005	0.0001

Supplementary table 8. Bayesian Mediation Estimates for the Associations Between Thought Dimensions and Topographic Similarity Across Tapping Conditions.

**Condition**	**Thought dimension**	**Variable**	**Mean**	**q5**	**q95**	$\hat{r}$
Fast	Task-relatedness	Indirect	0.14	*0.08*	*0.20*	1.00
		Direct	-0.04	-0.12	0.04	1.00
		Total	0.10	*0.05*	*0.15*	1.00
	Orientation	Indirect	45.50	-43.90	137.00	1.07
		Direct	-45.40	-137.00	43.90	1.07
		Total	0.06	*0.01*	*0.11*	1.00
Slow	Task-relatedness	Indirect	2.01	-6.15	17.40	1.84
		Direct	-1.97	-17.30	6.19	1.84
		Total	0.04	*-0.01*	*0.09*	1.00
	Orientation	Indirect	0.01	*0.01*	*0.02*	1.00
		Direct	-0.01	-0.05	0.04	1.00
		Total	0.01	-0.04	0.06	1.00

Note: Effects whose 90% credible intervals do not include zero are interpreted as statistically meaningful (*italicized* in parentheses).

Supplementary table 9. r values of correlations among task-relatedness, its smooth term, and topographic similarity under the slow tapping condition.

	Task-relatedness	s(task-relatedness)	Topographic similarity
Task-relatedness	1.00	1.00	0.04
s(task-relatedness)	1.00	1.00	0.04
Topographic similarity	0.04	1.00	1.00

Supplementary table 10. Nonlinear Modulation of the Relationship Between Thought Dimensions and PE Across timescales (tapping conditions) on replication dataset

**Fast Tapping**					
**LMM raw values**					
Predictor	Beta	SE	t	CI_lower	CI_upper
Task relatedness	-0.0009	0.0002	-3.8930	-0.0013	-0.0004
Orientation	-0.0001	0.0002	-0.4151	-0.0005	0.0003
**GAM**					
Predictor	edf	F_value	p_value		
Task relatedness	2.2930	3.1067	0.0298		
Orientation	3.7478	3.2626	0.0102		
**LMM nonlinearity controlled**					
Predictor	Beta	SE	t	CI_lower	CI_upper
Task relatedness	-0.0003	0.0002	-1.2020	-0.0007	0.0002
Orientation	-0.0003	0.0002	-1.3340	-0.0007	0.0001
**Slow Tapping**					
**LMM raw values**					
Predictor	Beta	SE	t	CI_lower	CI_upper
Task relatedness	-0.0007	0.0002	-3.5314	-0.0011	-0.0003
Orientation	-0.0007	0.0002	-3.3478	-0.0011	-0.0003
**GAM**					
Predictor	edf	F_value	p_value		
Task relatedness	6.0999	7.0964	0.0000		
Orientation	1.0003	17.7928	0.0000		
**LMM nonlinearity controlled**					
Predictor	Beta	SE	t	CI_lower	CI_upper
Task relatedness	-0.0001	0.0002	-0.5041	-0.0005	0.0003
Orientation	0.0005	0.0002	2.6413	0.0001	0.0009

Supplementary table 11. Bayesian Mediation Estimates for the Associations Between Thought Dimensions and PE on replication dataset.

Condition	Thought dimension	Variable	Mean	q5	q95	$\hat{r}$
Fast	Task-relatedness	Indirect	-0.13	*-0.21*	*-0.04*	1.00
		Direct	0.03	-0.07	0.15	1.00
		Total	-0.09	*-0.16*	*-0.02*	1.00
	Orientation	Indirect	0.03	*0.02*	*0.06*	1.00
		Direct	-0.01	-0.08	0.06	1.00
		Total	0.03	-0.04	0.1	1.00
Slow	Task-relatedness	Indirect	-0.11	*-0.15*	*-0.08*	1.00
		Direct	0.01	-0.06	0.09	1.00
		Total	-0.1	-0.18	-0.03	1.00
	Orientation	Indirect	-0.54	-1.29	0.11	2.68
		Direct	0.35	-0.30	1.11	2.67
		Total	-0.19	-0.27	-0.12	1.01

Note: Effects whose 90% credible intervals do not include zero are interpreted as statistically meaningful (*italicized* in parentheses).

Supplementary table 12. r values of Pearson Correlations Among Orientation, Smooth Term s(orientation), and PE on replication dataset

	orientation	s(orientation)	PE
orientation	1.00	-1.00	-0.19
s(orientation)	-1.00	1.00	0.19
PE	-0.19	0.19	1.00

Supplementary table 13. Interaction GAM Analysis Comparing Nonlinear Contributions of Thought Dimensions on PE Across Conditions on replication dataset

Predictor	F	p
Task-Relatedness (T)	9.66	<0.0001
Orientation (O)	160.62	<0.0001

Supplementary table 14. Nonlinear modeling of the relationship between thought dimensions and topographic similarity across tapping conditions on replication dataset.

Fast Tapping					
**LMM raw values**					
Predictor	Beta	SE	t	CI_lower	CI_upper
Task relatedness	0.0006	0.0003	2.4048	0.0001	0.0011
Orientation	0.0002	0.0003	0.7144	-0.0003	0.0007
**GAM**					
Predictor	edf	F_value	p_value		
Task relatedness	4.3084	6.3314	0.0000		
Orientation	3.9575	5.7078	0.0001		
**LMM nonlinearity controlled**					
Predictor	Beta	SE	t	CI_lower	CI_upper
Task relatedness	-0.0002	0.0003	-0.8320	-0.0007	0.0003
Orientation	-0.0006	0.0003	-2.2302	-0.0011	-0.0001

Slow Tapping					
**LMM raw values**					
Predictor	Beta	SE	t	CI_lower	CI_upper
Task relatedness	0.0000	0.0003	-0.1181	-0.0006	0.0006
Orientation	0.0004	0.0003	1.1779	-0.0002	0.0010
**GAM**					
Predictor	edf	F_value	p_value		
Task relatedness	2.7999	1.7507	0.1655		
Orientation	4.1595	7.7648	0.0000		
**LMM nonlinearity controlled**					
Predictor	Beta	SE	t	CI_lower	CI_upper
Task relatedness	-0.0005	0.0003	-1.5519	-0.0011	0.0001
Orientation	-0.0007	0.0003	-2.1870	-0.0013	-0.0001

Supplementary table 15. Bayesian Mediation Estimates for the Associations Between Thought Dimensions and topographic similarity on replication dataset.

Condition	Thought dimension	Variable	Mean	q5	q95	$\hat{r}$
Fast	Task-relatedness	Indirect	0.19	*0.12*	*0.25*	1.00
		Direct	-0.03	-0.12	0.06	1.00
		Total	0.16	*0.09*	*0.23*	1.00
	Orientation	Indirect	0.18	*0.12*	*0.25*	1.00
		Direct	-0.03	-0.13	0.07	1.00
		Total	0.15	*0.08*	*0.22*	1.00
Slow	Task-relatedness	Indirect	0.14	*0.05*	*0.22*	1.00
		Direct	-0.07	-0.18	0.04	1.00
		Total	0.07	-0.01	0.14	1.00
	Orientation	Indirect	0.21	*0.14*	*0.28*	1.00
		Direct	-0.03	-0.13	0.07	1.00
		Total	0.18	*0.11*	*0.26*	1.00

Note: Effects whose 90% credible intervals do not include zero are interpreted as statistically meaningful (*italicized* in parentheses).

References

1. A. K. Amrani, E. Z. Golumbic, Memory-paced tapping to auditory rhythms: Effects of rate, speech and motor-engagement. *bioRxiv* 2021.07.13.452153 (2021).

2. D. Rose, D. J. Cameron, P. J. Lovatt, J. A. Grahn, L. E. Annett, Comparison of spontaneous motor tempo during finger tapping, toe tapping and stepping on the spot in people with and without parkinson's disease. *J. Mov. Disord.* **13**, 47–56 (2020).

3. K. Christoff, A. M. Gordon, J. Smallwood, R. Smith, J. W. Schooler, Experience sampling during fMRI reveals default network and executive system contributions to mind wandering. *Proc. Natl. Acad. Sci.* **106**, 8719–8724 (2009).

4. M. S. Franklin, J. Smallwood, J. W. Schooler, Catching the mind in flight: Using behavioral indices to detect mindless reading in real time. *Psychon. Bull. Rev.* **18**, 992–997 (2011).

5. J. Hua, *et al.*, Alpha and theta peak frequency track on- and off-thoughts. *Commun. Biol.* **5**, 1–13 (2022).

6. C. Mills, Q. Raffaelli, Z. C. Irving, D. Stan, K. Christoff, Is an off-task mind a freely-moving mind? Examining the relationship between different dimensions of thought. *Conscious. Cogn.* **58**, 20–33 (2018).

7. J. Smallwood, J. W. Schooler, The restless mind. *Psychol. Bull.* **132**, 946–958 (2006).

8. A. K. Amrani, E. Z. Golumbic, Spontaneous and stimulus-driven rhythmic behaviors in ADHD adults and controls. *Neuropsychologia* **146**, 107544 (2020).

9. A. Kliger Amrani, E. Zion Golumbic, Testing the stability of 'Default' motor and auditory-perceptual rhythms–A replication failure dataset. *Data Brief* **32**, 106044 (2020).

10. A. Delorme, S. Makeig, EEGLAB: an open source toolbox for analysis of single-trial EEG dynamics including independent component analysis. *J. Neurosci. Methods* **13**, 9–21 (2004).

11. I. Winkler, S. Haufe, M. Tangermann, Automatic Classification of Artifactual ICA-Components for Artifact Removal in EEG Signals. *Behav. Brain Funct.* **7**, 30 (2011).

12. I. Winkler, *et al.*, Robust artifactual independent component classification for BCI practitioners. *J. Neural Eng.* **11**, 035013 (2014).

13. M. Luo, H. Zhang, H. Luo, Reactivated past decisions repel early sensory processing and attract late decision-making. [Preprint] (2024). Available at: http://biorxiv.org/lookup/doi/10.1101/2024.02.26.582221 [Accessed 16 August 2024].

14. X. Tian, D. E. Huber, Measures of Spatial Similarity and Response Magnitude in MEG and Scalp EEG. *Brain Topogr.* **20**, 131–141 (2008).

15. J.-P. Lachaux, E. Rodriguez, J. Martinerie, F. J. Varela, Measuring phase synchrony in brain signals. *Hum. Brain Mapp.* **8**, 194–208 (1999).

16. S. Lechner, G. Northoff, Temporal imprecision and phase instability in schizophrenia resting state EEG. *Asian J. Psychiatry* **86**, 103654 (2023).

17. J. Theiler, S. Eubank, A. Longtin, B. Galdrikian, J. Doyne Farmer, Testing for nonlinearity in time series: the method of surrogate data. *Phys. Nonlinear Phenom.* **58**, 77–94 (1992).

18. A. V. Bocharov, G. G. Knyazev, A. N. Savostyanov, T. N. Astakhova, S. S. Tamozhnikov, EEG dynamics of spontaneous stimulus-independent thoughts. *Cogn. Neurosci.* **10**, 77–87 (2019).

19. A. Wolff, J. Gomez-Pilar, T. Nakao, G. Northoff, Interindividual neural differences in moral decision-making are mediated by alpha power and delta/theta phase coherence. *Sci. Rep.* **9**, 1–13 (2019).

20. E. Barron, L. M. Riby, J. Greer, J. Smallwood, Absorbed in Thought: The Effect of Mind Wandering on the Processing of Relevant and Irrelevant Events. *Psychol. Sci.* **22**, 596–601 (2011).

21. J. Polich, *Theoretical Overview of P3a and P3b* (Springer, 2003).

22. J. Polanco-Martinez M., M. Medina-Elizalde A., M. Goni Fernanda,Sanchez, M. Mudelsee, BINCOR: An R package for Estimating the Correlation between Two Unevenly Spaced Time Series. *R J.* **11**, 170 (2019).

23. S. Taheri-Araghi, *et al.*, Cell-Size Control and Homeostasis in Bacteria. *Curr. Biol.* **25**, 385–391 (2015).

24. R. Frömer, M. Maier, R. Abdel Rahman, Group-level EEG-processing pipeline for flexible single trial-based analyses including linear mixed models. *Front. Neurosci.* **12**, 1–15 (2018).

25. Z. Huang, *et al.*, Is There a Nonadditive Interaction Between Spontaneous and Evoked Activity? Phase-Dependence and Its Relation to the Temporal Structure of Scale-Free Brain Activity. *Cereb. Cortex N. Y. N 1991* **27**, 1037–1059 (2017).

26. G. Northoff, N. W. Duncan, D. J. Hayes, The brain and its resting state activity—Experimental and methodological implications. *Prog. Neurobiol.* **92**, 593–600 (2010).

27. G. Northoff, D. Vatansever, A. Scalabrini, E. A. Stamatakis, Ongoing Brain Activity and Its Role in Cognition: Dual versus Baseline Models. *The Neuroscientist* 1–28 (2022). https://doi.org/10.1177/10738584221081752.

28. R. L. Buckner, L. M. DiNicola, The brain's default network: updated anatomy, physiology and evolving insights. *Nat. Rev. Neurosci.* **20**, 593–608 (2019).

29. V. Menon, 20 years of the default mode network: A review and synthesis. *Neuron* **111**,

2469–2487 (2023).

30. E. Maris, R. Oostenveld, Nonparametric statistical testing of EEG- and MEG-data. *J. Neurosci. Methods* **164**, 177–190 (2007).

31. S. N. Wood, *Generalized Additive Models: An Introduction with R*, 2nd Ed. (Chapman and Hall/CRC, 2017).

32. G. Northoff, F. Zilio, J. Zhang, Beyond task response—Pre-stimulus activity modulates contents of consciousness. *Phys. Life Rev.* **49**, 19–37 (2024).
